# The Relationship Between Gut Microbiota During Pregnancy and the Level of Postpartum Adiposity

**DOI:** 10.1002/mbo3.70128

**Published:** 2025-11-12

**Authors:** Noora Houttu, Kati Mokkala, Himmi Lindgren, Mrunalini Lotankar, Chouaib Benchraka, Katariina Pärnänen, Lotta Saros, Ella Muhli, Tero Vahlberg, Leo Lahti, Kirsi Laitinen

**Affiliations:** ^1^ Integrative Physiology and Pharmacology Unit, Institute of Biomedicine University of Turku Turku Finland; ^2^ Nutrition and Food Research Center University of Turku Turku Finland; ^3^ Department of Computing, Faculty of Technology University of Turku Turku Finland; ^4^ Department of Microbiology, Faculty of Agriculture and Forestry University of Helsinki Helsinki Finland; ^5^ Department of Obstetrics and Gynecology University of Turku Turku Finland; ^6^ Department of Pediatrics Turku University Hospital Turku Finland; ^7^ Department of Biostatistics University of Turku and Turku University Hospital Turku Finland; ^8^ Department of Obstetrics and Gynecology Turku University Hospital, Wellbeing Services County of Southwest Finland Turku Finland

**Keywords:** gut microbiota, metagenomics, obesity, postpartum, pregnancy

## Abstract

Gut microbiota is linked with health, including obesity, in the general population. It is unknown whether adiposity at postpartum is influenced by gut microbiota already during pregnancy. We investigated the association between the gut microbiota's composition and predicted function by metagenomics during pregnancy and the women's adiposity (body mass index [BMI], waist‐to‐hip ratio [WHR], body fat%) assessed at 1‐, 2‐, and 5–6‐years' postpartum in 257 women with overweight or obesity based on prepregnancy BMI values. Body fat% at 1‐year, but not at 2‐ or 5–6‐years' postpartum, was associated inversely with *α*‐diversity during pregnancy. Bacterial species GGB3034 SGB4030 (family *Erysipelotrichaceae*) was higher in women with normal weight than those in women with obesity at 1‐year postpartum (*q* = 0.02), other species being borderline statistically significant (*q* < 0.25). High WHR and body fat% at 1‐year postpartum were associated with two species (*q* < 0.25). Considering predicted functions of bacteria, an association was detected for BMI, WHR, and body fat%, e.g., body fat% and glycogen biosynthesis I (*q* < 0.25). Gut microbiota during pregnancy predicted the BMI and body fat% at 1‐year postpartum (ROC > 0.50, *p* < 0.02). Postpartum adiposity was associated with several species and *α*‐diversity. Gut microbiota during pregnancy may be involved in the persistence of obesity and its comorbidities after pregnancy.

## Graphical abstract

Postpartum adiposity is associated with maternal *α*‐diversity and several bacterial species. These findings highlight the mediating role of the gut microbiota during pregnancy in influencing the overweight and obesity condition postpartum. Created in BioRender. Mokkala, K. (2025) https://BioRender.com/l8v4f0i

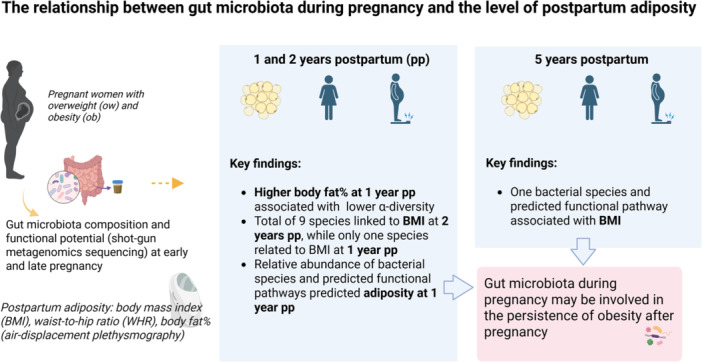

## 1 Introduction

Obesity is an increasing global threat since it is linked with a multitude of comorbidities. The prevalence of pregnant women living with overweight or obesity (determined by body mass index [BMI] equal or greater than 25 or 30 kg/m^2^, respectively, measured before pregnancy or at early pregnancy) had risen to affect almost every second woman on a global scale in the last decade and it is estimated that this value will increase by 0.64% annually (Kent et al. 2024). As obesity during pregnancy increases the health risks for the mother and also for her child in later life, it is crucial to determine which of the obesity associated factors contribute to the postpartum health and thus to develop effective means to intervene. Gut microbiota has been identified as one such factor.

Previous studies with pregnant women have revealed differing gut microbiota compositions according to the woman's overweight and obesity status and recommended weight gain during pregnancy (Collado et al. 2008; Dreisbach et al. 2020; Houttu et al. 2023; Santacruz et al. 2010). Some studies have hinted that there may be a correlation between gut microbiota and the incidence of gestational diabetes mellitus (GDM) (Cortez et al. 2019; Crusell et al. 2018; Hu et al. 2021; Ma et al. 2020), but thus far, the findings have been inconsistent (Mokkala et al. 2021). We have shown that the gut microbiota during pregnancy is linked with prediabetes status at 2‐years' postpartum (Houttu et al. 2023), however, as far as we are aware, no studies exist that would have examined the relationship of the gut microbiota during pregnancy to the occurrence of postpartum adiposity.

Thus, we conducted a study in which we hypothesized that the particular gut microbiota during pregnancy could be linked to adiposity over a 5–6‐years' postpartum period. The aim was to investigate the relationship of the composition and function of the gut microbiota in early and late pregnancy, as well as its change between these two time points to the women's adiposity assessed at 1‐, 2‐, and 5–6‐years' postpartum. In more detail, we assessed if the adiposity levels were involved by (1) investigating the relationship of gut microbiota in pregnancy to the adiposity measures of women (waist‐to‐hip ratio [WHR], BMI, fat%, and body fat% trajectories), (2) comparing if there were differences in the gut microbiota in pregnancy between women with normal weight, overweight, and obesity, and between women with normal and high WHR at several postpartum assessments, and finally (3) evaluating whether it would be possible to predict the postpartum adiposity by determining the gut microbiota present during pregnancy.

## 2 Materials and Methods

### 2.1 Study Design and Patients

The study subjects (in total, *n* = 439) in this mother–child clinical trial (ClinicalTrials.gov: NCT01922791) were recruited in Turku, Southwest Finland in 10/2013–7/2017. The inclusion criteria were overweight (prepregnancy BMI ≥ 25 kg/m^2^) and early pregnancy (< 18 gestational weeks). The exclusion criteria were diabetes before pregnancy, multifetal pregnancy and chronic diseases impacting metabolic and gastrointestinal health, including inflammatory bowel diseases. The design and participants have been described in detail previously (Pellonperä et al. 2019). The main trial aims to investigate the health effects of fish oil and/or probiotics in women with overweight and obesity and their children. Here, the intervention is included as a covariate. Furthermore, while the intervention effect was evaluated regarding BMI, WHR, and body fat% at 1‐, 2‐, and 5–6‐years' postpartum, it was observed that it did not have any effect on the adiposity measures (data not shown).

This secondary analysis of the main trial aimed to investigate the relationship of the gut microbiota in early and late pregnancy to adiposity assessed in mothers at 1‐, 2‐, and 5–6‐years' postpartum. Therefore, those women who had used antibiotics in early (*n* = 28) and late pregnancy (*n* = 26) and women who had delivered another child, had experienced a miscarriage or were pregnant at the postpartum assessment (*n* = 39), were excluded. In addition, we did not include those women who had used insulin or metformin medication in late pregnancy (*n* = 22). This resulted in 255 women with fecal samples being analyzed both in early (mean 14.0 SD 2.0) and late (mean 35.2 SD 1.0) pregnancy.

### 2.2 Measures of Adiposity

The women's adiposity was assessed by using three measures: BMI, WHR, and fat% at 1‐, 2‐, and 5–6‐years' postpartum. BMI was calculated by dividing the weight (kg) by height (m) squared. Weight was measured at each postpartum study visit with a calibrated electronic scale (the Bod Pod system, software version 5.4.0, COSMED Inc.) and height with a wall stadiometer to the nearest 0.1 cm at the early pregnancy study visit. Normal weight was defined as BMI 18.5–24.99 kg/m^2^, overweight as BMI 25–29.99 kg/m^2^ and obesity as BMI ≥ 30 kg/m^2^. Waist and hip circumference (cm) were measured with a measuring tape (Seca Measuring Tape 201, Seca GmbH & Co. KG, 22089 Hamburg, Germany), and WHR was calculated by dividing waist circumference by hip circumference. WHR < 0.85 was defined as normal and WHR ≥ 0.85 as high, according to the expert consultation on WHR issued by the World Health Organization (WHO 2011). The fat% was measured with air displacement plethysmography (the Bod Pod system, software version 5.4.0, COSMED Inc.) with the Siri equation (Siri 1993) at 3‐ and 6‐months' and 1‐, 2‐, and 5–6‐years' postpartum.

LGMM in SAS 9.4 (SAS Institute Inc. Cary, NC) was used to model trajectories of body fat%, from 3‐ to 24‐months' postpartum as described by Muhli et al. (2025). The number of latent growth curves was established by increasing the number of groups in LGMM and comparing the fit indices of the models. A posterior group probability (with respect to the probability of an individual belonging to a trajectory group; a score of > 0.80 was preferred), the class size (the classes that were identified should not comprise less than 5% of the sample), and clinical interpretability were used to decide the optimal number of trajectory groups. Two trajectories were identified: (1) body fat% decreased first and then rose slowly, that is, decreasing and slowly rising group and (2) body fat% was high and stable, that is, high and stable group (Log. L, −3185.88; AIC, −3193.88; BIC, −3209.06; group membership, 48.5%/51.5%; average group posterior probabilities, 0.95/0.93).

### 2.3 Other Clinical Parameters

Fasting (at least 9 h) blood samples were drawn from each woman's antecubital vein in the morning of the early pregnancy study visit. Fasting plasma levels of insulin and glucose were determined with an immunoelectrochemiluminometric assay on a modular E170 automatic analyzer (Roche Diagnostics GmbH, Mannheim, Germany) and an enzymatic method utilizing hexokinase (Cobas 8000 automatic c702‐analyzer, Roche Diagnostics GmbH, Mannheim, Germany), respectively. High‐sensitivity C‐reactive protein was analyzed by an automated colorimetric immunoassay on the Dade Behring Dimension RXL autoanalyzer (Siemens Healthcare, Camberly, Surrey, UK).

Diastolic and systolic blood pressure values of the women were measured with Omron M5‐1 (Intelli sense, Omron Matsusaka Co. Ltd. Japan).

Prepregnancy BMI was calculated by dividing the self‐reported weight (kg), collected at maternal welfare clinic records, by height (m), measured at the early pregnancy study visit, squared. The daily dietary information of the women was collected at the early pregnancy study visit with 3‐day food diaries (two weekdays and one weekend day) in the week preceding the study visit, and the diaries were checked by the study coordinator at the study visit. The daily dietary intakes of energy, carbohydrates, protein, fat, polyunsaturated fatty acids (PUFAs), monosaturated fatty acids (MUFAs), and saturated fatty acids (SFAs) as grams and energy percent were calculated by software (AivoDiet 2.0.2.3; Aivo, Turku, Finland) utilizing the food composition database Fineli.

### 2.4 Fecal Sampling and Gut Microbiota Analysis

The women's fecal samples were collected in plastic pots at home on the previous evening or on the same morning of the study visit in early and late pregnancy and kept at −20°C until DNA extraction, which was performed in the Department of Clinical Microbiology and Immunology, University of Turku (Finland). The metagenomics sequencing was done with the Illumina HiSeq‐platform using paired‐end sequencing in Clinical Microbiomics (Denmark). The DNA extraction, sequencing, quality control, and removal of host sequences have been described in detail previously (Mokkala et al. 2021).

A taxonomic classification was performed using MetaPhlan v4.0.1 (Blanco‐Míguez et al. 2023) with a database spanning 26,970 species‐level genome bins (Pasolli et al. 2019) using all default settings. Functional profiles were obtained using HUMAnN v3.0.1 (Beghini et al. 2021) using the full pangenome and protein (UniRef90) database as well as with the default settings. Both analyses were performed using a distributed cloud computing environment (CSC) with Snakemake v7.6.1 (Mölder et al. 2021).

### 2.5 Data Analysis

The downstream data analysis was performed using R version 4.4.1. The data were contained in a MultiAssayExperiment infrastructure (Ramos et al. 2017).

The differences in early pregnancy characteristics with regard to the adiposity variables were tested using Kruskal–Wallis and Wilcoxon rank‐sum test for continuous outcome variables, whereas the summary statistics for categorical variables were calculated separately for each outcome level using Fisher's exact test and Pearson's Chi‐squared test. Benjamini–Hochberg (BH) correction was used to control the False Discovery Rate (FDR), which was calculated based on the number of clinical characteristics.

The following variables were included as covariates: intervention group, early pregnancy fasting plasma insulin concentration, and early pregnancy daily dietary intake of fat. Fasting plasma insulin and intake of fat differed between the groups (Table 1) and thus might have influenced the analyses. The other variables that differed between the groups were not included as covariates because they correlated statistically significantly with fasting plasma insulin and fat intake (data not shown).

We analyzed community diversity at the species level within samples (*α*‐diversity; Shannon index) and between‐sample variation in community composition (*β*‐diversity). The analyses were evaluated separately by linear regression in early and late pregnancy, comparing across outcome groups, as well as examining changes from early to late pregnancy. Sample pairing was considered when evaluating changes over time. Differences in community composition (*β*‐diversity) were evaluated with PERMANOVA tests that were conducted separately at each time point; this was also undertaken with respect to the changes over time, as a way to evaluate the overall differences in microbial composition with respect to the adiposity variables. The covariates included intervention, early pregnancy fasting plasma insulin and early pregnancy daily dietary intake of fat. In the models, the outcome variable was community diversity, with the exploratory variables being the adiposity variables. The homogeneity of the groups was tested with *β*‐dispersion before PERMANOVA. The *β*‐diversity was visualized using Principal Coordinate Analysis (PCoA), where the samples were reflected onto two‐dimensional space calculated using Bray–Curtis dissimilarity index.

A differential abundance analysis was undertaken to identify the individual taxa that displayed differential abundances associated with the adiposity markers. The differential abundance analysis was performed using multivariate analysis by linear models available in the MaAsLin2 R package, and conducted for early and late pregnancy separately, as well as for changes between these periods, as well as for functional pathways. Overall, 1385 rare species were filtered out based on a 10% prevalence at the 0.01% detection thresholds on relative abundance data, resulting in 302 bacterial species (Table S1). The same filtering thresholds were applied to the pathways, reducing the original value of 579 pathways down to 372. BH correction was used to control the FDR, which was calculated based on the number of taxonomic or functional features. The models included intervention, early pregnancy fasting plasma insulin levels and early pregnancy daily dietary intake of fat as covariates.

We applied Random Forests to assess the predictive performance of adiposity markers with early, late values, and the change from early to late pregnancy in the relative abundance of bacterial species and predicted functional pathways. For this, a 10% prevalence at the 0.01% detection threshold was applied for taxonomic and functional data, resulting in the inclusion of 302 bacterial species and 372 pathways. The taxonomic data were transformed using a Centered Log‐Ratio transformation. The model's performance was assessed with 10‐fold cross‐validation, where each 90% subset of the data was split again into 30%/70% testing and training sets. For each training set, we trained both a Random Forest and random baseline models, where the random baseline was constructed using shuffled levels of the response variable. The significance of the predictive performance of the Random Forest was assessed using the Wilcoxon rank‐sum test, which compared the performance of the predictive models against random baseline models, with the AUROC value representing an indicator of performance. Permutation importance was used to define the key features regarding the predictive performance, and the overall importance combined the most important features of all 10 folds. The models included prevalent taxa or functional features, covariates, and a combined set of both.

The statistically significant findings are depicted with a *p* < 0.05 or FDR‐adjusted *p* values, that is, a *q* < 0.05 and the borderline statistically significant findings with a *q* < 0.25.

## 3 Results

### 3.1 Clinical Characteristics of the Women

The early pregnancy clinical characteristics of the women with respect to their adiposity measures at postpartum are presented in Tables 1 and S2–S8. Most of the differences in clinical characteristics were detected between BMI groups at 2‐years' postpartum, whilst at other time points, no differences were found (*q* < 0.05, Table 1). Most of the women with obesity before pregnancy (prepregnancy BMI ≥ 30 kg/m^2^) were also living with obesity at 1‐, 2‐, and 5–6‐years' postpartum (86%, 90%, and 83%, respectively). Two‐thirds of the women with overweight (prepregnancy BMI 25–29.99 kg/m^2^) were living with overweight at 1‐year (63%) and 2 years' postpartum (61%), while at 5–6 years, rather many of these women were classified in the obesity group (44% vs. 43% with overweight). About every tenth (8%–11%) of the women were living with normal weight at all postpartum time points (Figure S1a), all of those had lived with overweight before pregnancy and none with obesity (Table 1 and Figure S1b). The women with higher measures of adiposity at 1‐year postpartum also exhibited higher values of both blood pressure and fasting plasma insulin at early pregnancy. Higher early pregnancy blood pressure and fasting insulin levels were detected in women with obesity at 2‐year postpartum assessment, while only the fasting insulin level was higher in women with obesity at 5–6 years. The intakes of total fat, PUFAs, MUFAs, and SFAs in grams in early pregnancy were higher in women with overweight as compared with women with normal weight or obesity at 2‐year postpartum. The other assessed characteristics, including age, education, parity, ethnicity, smoking before pregnancy, gestational weight gain, or other nutrients, did not differ between the groups.

**Table 1:** Clinical characteristics of pregnant women at early pregnancy according to their normal‐weight/overweight/obesity status at 2‐years' postpartum.

BMI at 2‐years' postpartum	All^a^ *N* = 174	Normal weight^a^ *N* = 12	Overweight^a^ *N* = 69	Obese^a^ *N* = 93	*p* value^b^	*q* value^c^
Age (years)	31.7 (28.6, 35.1)	32.2 (28.5, 34.7)	32.0 (29.2, 34.8)	31.6 (28.4, 35.2)	> 0.9	> 0.9
College or university education (*n*, %)	116 (66.7)	10 (83.3)	48 (69.6)	58 (62.4)	0.3	0.6
Smoked before pregnancy (*n*, %)	33 (19.0)	3 (25.0)	11 (15.9)	19 (20.4)	0.6	0.8
Prepregnancy BMI (kg/m^2^)	29.1 (26.5, 32.0)	24.9 (24.5, 25.5)	26.8 (25.9, 28.3)	31.4 (29.5, 34.1)	< 0.001	< 0.001
Prepregnancy BMI (overweight or obese) (*n*, %)					< 0.001	< 0.001
Overweight	101 (58.0)	12 (100.0)	62 (89.9)	27 (29.0)		
Obese	73 (42.0)	0 (0.0)	7 (10.1)	66 (71.0)		
*Region of origin (n, %)*					0.6	0.8
European	171 (98.3)	12 (100.0)	69 (100.0)	90 (96.8)		
Asian	1 (0.6)	0 (0.0)	0 (0.0)	1 (1.1)		
Middle Eastern	0 (0.0)	0 (0.0)	0 (0.0)	0 (0.0)		
Other/mixed	2 (1.1)	0 (0.0)	0 (0.0)	2 (2.2)		
Primiparous (*n*, %)	75 (43.1)	5 (41.7)	24 (34.8)	46 (49.5)	0.2	0.4
Gestational weight gain from prepregnancy to late pregnancy (kg)	12.9 (8.8, 16.2)	11.6 (9.4, 13.2)	14.1 (10.7, 17.3)	12.2 (7.7, 16.0)	0.03	0.1
Blood pressure systolic (mmHg)	117.5 (111.0, 124.0)^d^	108.5 (106.0, 111.8)	115.5 (109.8, 121.0)^d^	119.0 (114.0, 127.0)	< 0.001	< 0.001
Blood pressure diastolic (mmHg)	78.0 (71.5, 83.0)^d^	73.3 (68.3, 76.0)	75.0 (70.3, 80.3)^d^	80.0 (73.5, 85.5)	< 0.001	0.004
Fasting glucose (mmol/L)	4.7 (4.5, 4.9)^d^	4.7 (4.6, 5.0)	4.7 (4.5, 4.9)^d^	4.8 (4.5, 5.0)^d^	0.4	0.6
Fasting insulin level (mU/L)	10.0 (7.0, 14.0)^d^	8.5 (7.5, 10.5)	8.0 (7.0, 10.0)^d^	12.0 (8.0, 16.0)^d^	< 0.001	0.004
GDM either in pregnancy (*n*, %)	47 (27.8)^d^	2 (16.7)	12 (17.4)	33 (37.5)^d^	0.01	0.07
High‐sensitivity CRP (mg/L)	5.7 (3.5, 8.8)^d^	4.0 (2.8, 6.6)	5.7 (3.2, 7.9)^d^	6.1 (3.6, 9.9)^d^	0.2	0.5
*Diet*						
Energy intake (kJ)	8251.5 (6897.5, 9868.0)^d^	7424.0 (6947.0, 8576.0)^d^	8630.0 (7543.0, 10335.0)	7943.0 (6555.0, 9185.0)^d^	0.02	0.09
Carbohydrate (E%)	45.0 (41.4, 49.0)^d^	44.7 (39.8, 51.4)^d^	44.2 (41.3, 47.2)	45.7 (41.6, 49.7)^d^	0.5	0.7
Protein (E%)	16.2 (14.1, 18.5)^d^	15.5 (13.2, 17.8)^d^	15.6 (13.9, 17.6)	17.1 (14.4, 18.6)^d^	0.2	0.4
Fat (E%)	36.6 (31.6, 40.1)^d^	36.3 (32.7, 41.4)^d^	37.2 (33.6, 40.2)	35.7 (31.0, 39.2)^d^	0.2	0.4
Polyunsaturated fatty acids (E%)	5.6 (4.6, 6.6)^d^	6.3 (4.6, 8.1)^d^	5.9 (4.9, 6.8)	5.3 (4.4, 6.3)^d^	0.04	0.2
Monounsaturated fatty acids (E%)	12.3 (10.3, 13.9)^d^	13.0 (10.6, 13.9)^d^	12.6 (11.2, 14.3)	11.9 (9.9, 13.7)^d^	0.06	0.2
Saturated fatty acids (E%)	12.9 (11.3, 14.9)^d^	12.6 (10.9, 12.9)^d^	13.5 (11.5, 15.4)	12.8 (11.3, 14.7)^d^	0.2	0.5
Carbohydrate intake (g)	218.0 (172.7, 267.8)^d^	213.6 (173.6, 235.3)^d^	224.7 (186.0, 271.9)	207.0 (165.6, 265.5)^d^	0.2	0.5
Protein (g)	78.6 (65.7, 93.6)^d^	68.9 (65.6, 85.7)^d^	78.7 (68.2, 96.3)	79.9 (64.7, 91.3)^d^	0.3	0.5
Fat (g)	81.1 (63.8, 97.9)^d^	74.7 (57.5, 95.9)^d^	88.6 (68.2, 109.2)	76.7 (60.3, 88.3)^d^	0.005	0.04
Polyunsaturated fatty acids (g)	12.6 (8.8, 16.8)^d^	12.8 (8.4, 19.6)^d^	14.2 (10.6, 17.9)	11.2 (8.2, 15.1)^d^	0.007	0.04
Monounsaturated fatty acids (g)	26.9 (21.0, 34.2)^d^	25.2 (20.8, 32.6)^d^	31.5 (24.1, 39.8)	25.7 (20.2, 30.6)^d^	0.003	0.03
Saturated fatty acids (g)	28.9 (22.8, 35.4)^d^	26.2 (23.6, 30.3)^d^	31.6 (26.2, 40.8)	27.3 (21.7, 34.2)^d^	0.006	0.04
Fiber (g)	20.5 (15.9, 26.2)^d^	20.0 (18.0, 27.4)^d^	22.7 (17.9, 27.1)	17.9 (14.4, 24.9)^d^	0.02	0.08

Abbreviations: BMI, body mass index; CRP, C‐reactive protein; GDM, gestational diabetes mellitus.

^a^ Median (Q1, Q3), *n* (%).

^b^ Kruskal–Wallis, Fisher's exact test, and Pearson's Chi‐squared test.

^c^ Benjamini–Hochberg correction was used to control the False Discovery Rate, which was calculated based on the number of clinical characteristics.

^d^ Missing values 1–6.

### 3.2 The Relationship of BMI, WHR, and Body Fat% at 1‐, 2‐, and 5–6‐Years' Postpartum on the Composition of the Gut Microbiota and Predicted Functional Pathways During Pregnancy

#### 3.2.1 *α*‐Diversity

**Figure 1:**
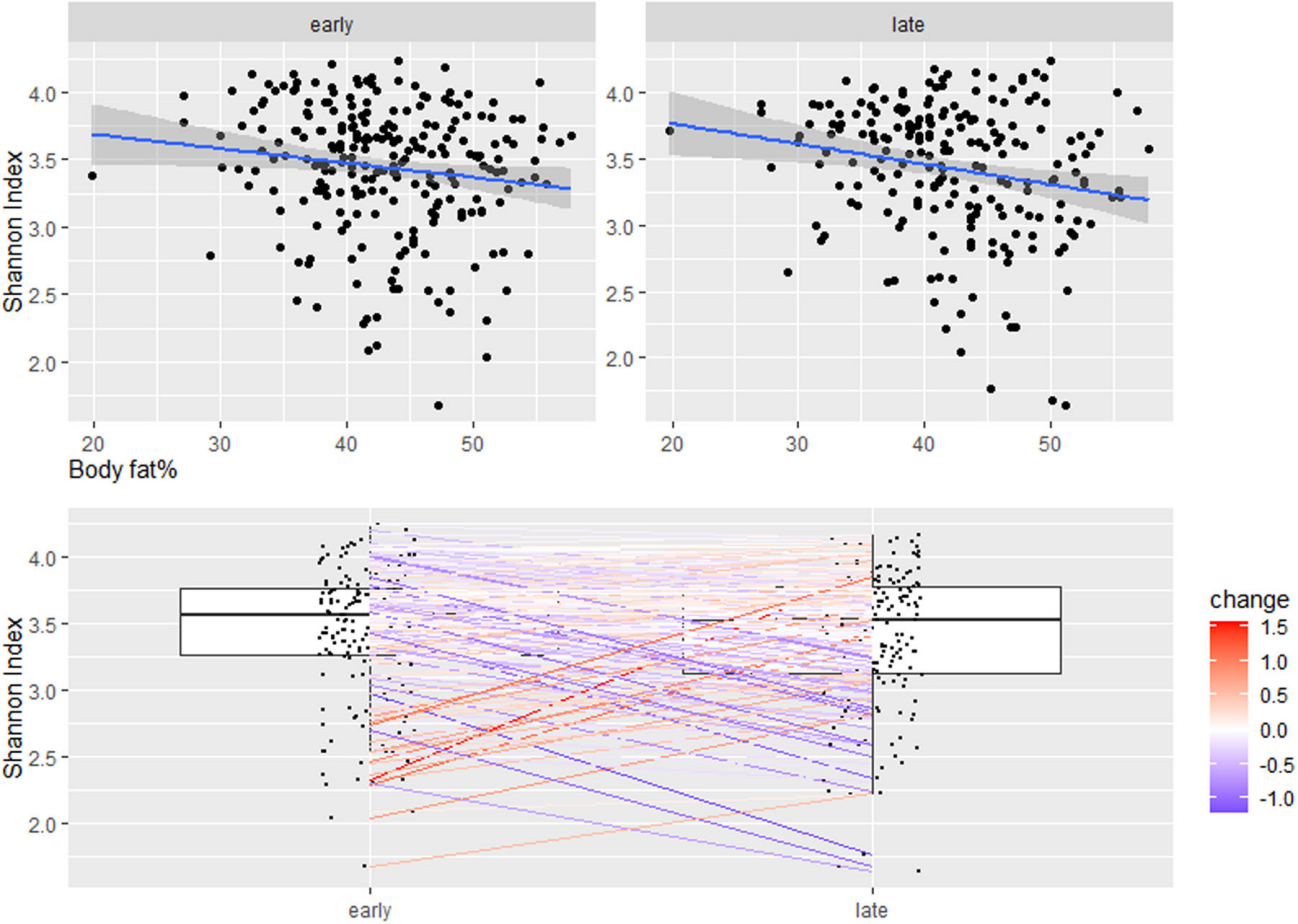
Body fat% at 1‐year postpartum associated with *α*‐diversity (Shannon index) in late pregnancy and the change from early to late pregnancy (*p* = 0.01 for both, MaAsLin2), but not in early pregnancy. The color indicates the degree of change between the time points. The statistically significant findings are depicted with a *p* < 0.05.

**Figure 2:**
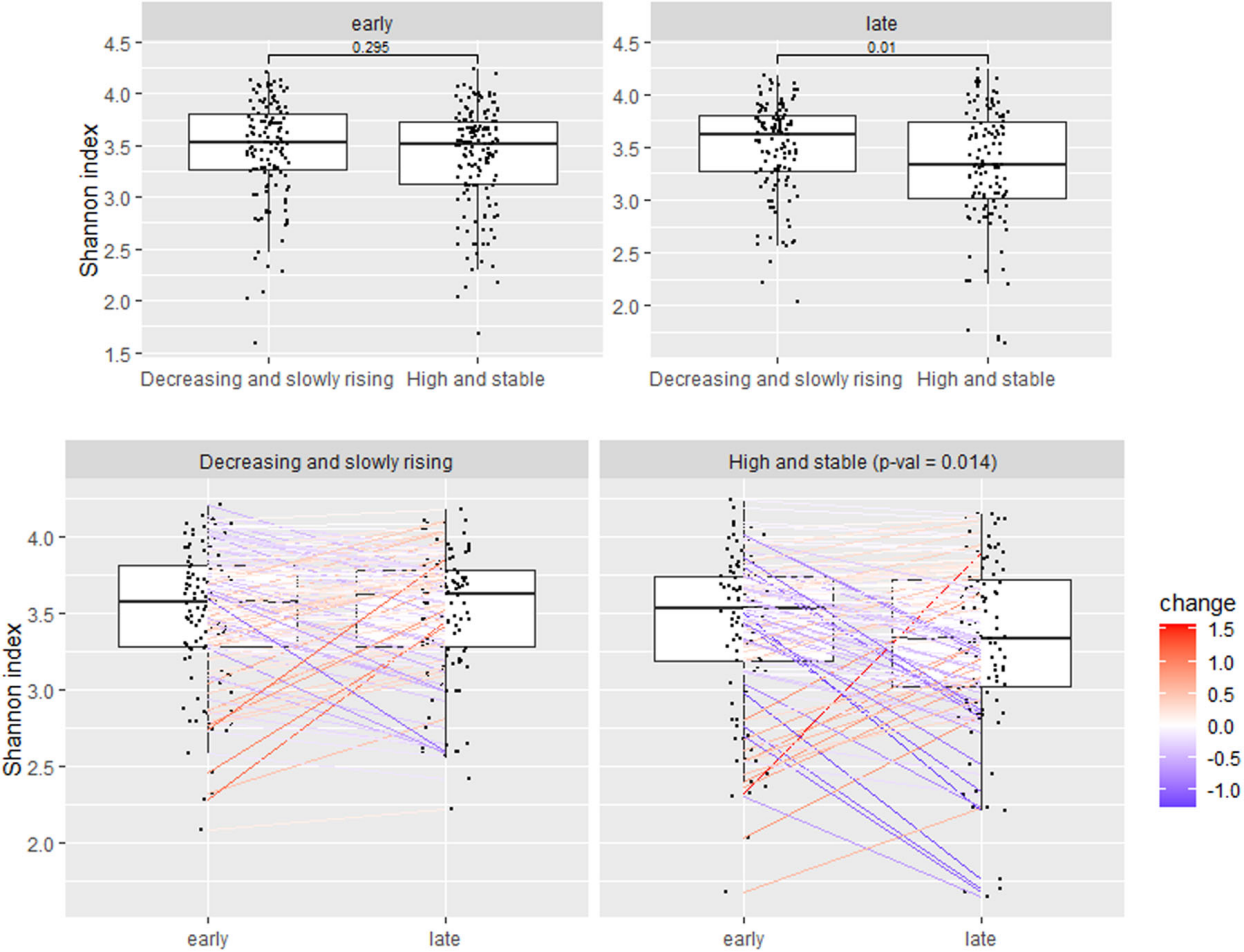
*α*‐Diversity (Shannon index) in late pregnancy was higher in those women who belonged to the decreasing and slowly rising group as compared with women in the high and stable group (*p* = 0.01, MaAsLin2). *α*‐Diversity (Shannon index) decreased from early to late pregnancy more in the high and stable group as compared with those in the decreasing and slowly rising group (*p* = 0.01, MaAsLin2). The color indicates the degree of change between the time points. The statistically significant findings are depicted with a *p* < 0.05.

*α*‐Diversity associated with body fat% at 1‐year postpartum (analyzed only as a continuous variable) in late pregnancy and change from early to late pregnancy, but not in early pregnancy (*β* −0.01, 95% CI −0.03 to 0.003, *p* = 0.01 and *β* −0.01, 95% CI −0.02 to 0.003, *p* = 0.01, respectively, Figure 1). The results were consistent with the body fat% trajectories (two trajectories from 3 to 24 months were identified: (1) body fat% decreased first and then rose slowly, that is, decreasing and slowly rising group and (2) body fat% was high and stable, that is, high and stable group). The *α*‐diversity at late pregnancy was lower in those women who belonged to the high and stable group as compared with women in the decreasing and slowly rising group (*β* −0.17, 95% CI −0.30 to −0.0.4, *p* = 0.01, Figure 2). Further, in the high and stable group, *α*‐diversity decreased statistically significantly more from early to late pregnancy as compared with the situation in the women in the decreasing and slowly rising group (*β* −0.15, 95% CI −0.26 to −0.03, *p* = 0.01, Figure 2). No associations were observed between any of the other adiposity markers and *α*‐diversity.

#### 3.2.2 *β*‐Diversity

No distinct clustering in the adiposity markers was visible in ordination (PCoA, Figures S2–S4). However, BMI (continuous) at 2‐ and 1‐year postpartum was associated with *β*‐diversity in early and late pregnancy (*p* = 0.04 and 0.045, respectively, PERMANOVA, Figure S2a,b). Moreover, body fat% at 1‐year postpartum was associated with *β*‐diversity in early and late pregnancy (*p* = 0.02 and 0.006, Figure S3) but not at the other postpartum time points. In addition, community composition in late pregnancy differed between the two groups, that is, those with decreasing and slowly rising in comparison with the high and stable group of body fat% trajectories (*p* = 0.03, PERMANOVA) (Figure S4). Furthermore, no relationships were detected between normal weight, overweight, or obesity status or WHR at any postpartum time points, and community composition (early, late, and change from early to late pregnancy, (*p* > 0.08, PERMANOVA).

#### 3.2.3 Bacterial Species

**Figure 3:**
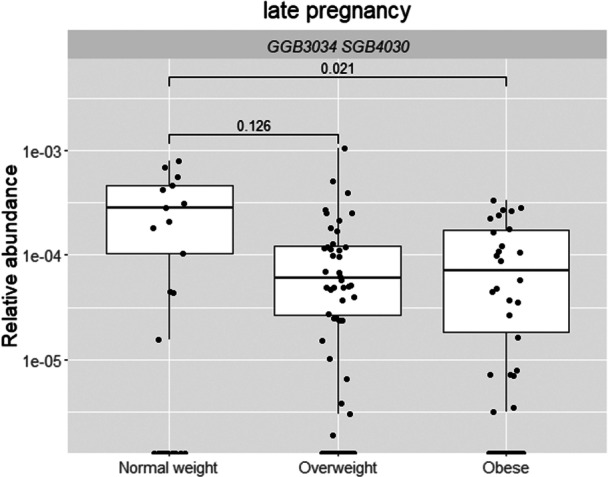
The relative abundance of GGB3034 SGB4030 (family *Erysipelotrichaceae*) in late pregnancy was higher in women with normal weight as compared with women with obesity (*q* = 0.02, MaAsLin2) at 1‐year postpartum. The statistically significant findings are depicted with an FDR‐adjusted *p* value, that is, *q* < 0.05 and the borderline statistically significant findings with a *q* < 0.25. FDR, False Discovery Rate.

**Figure 4:**
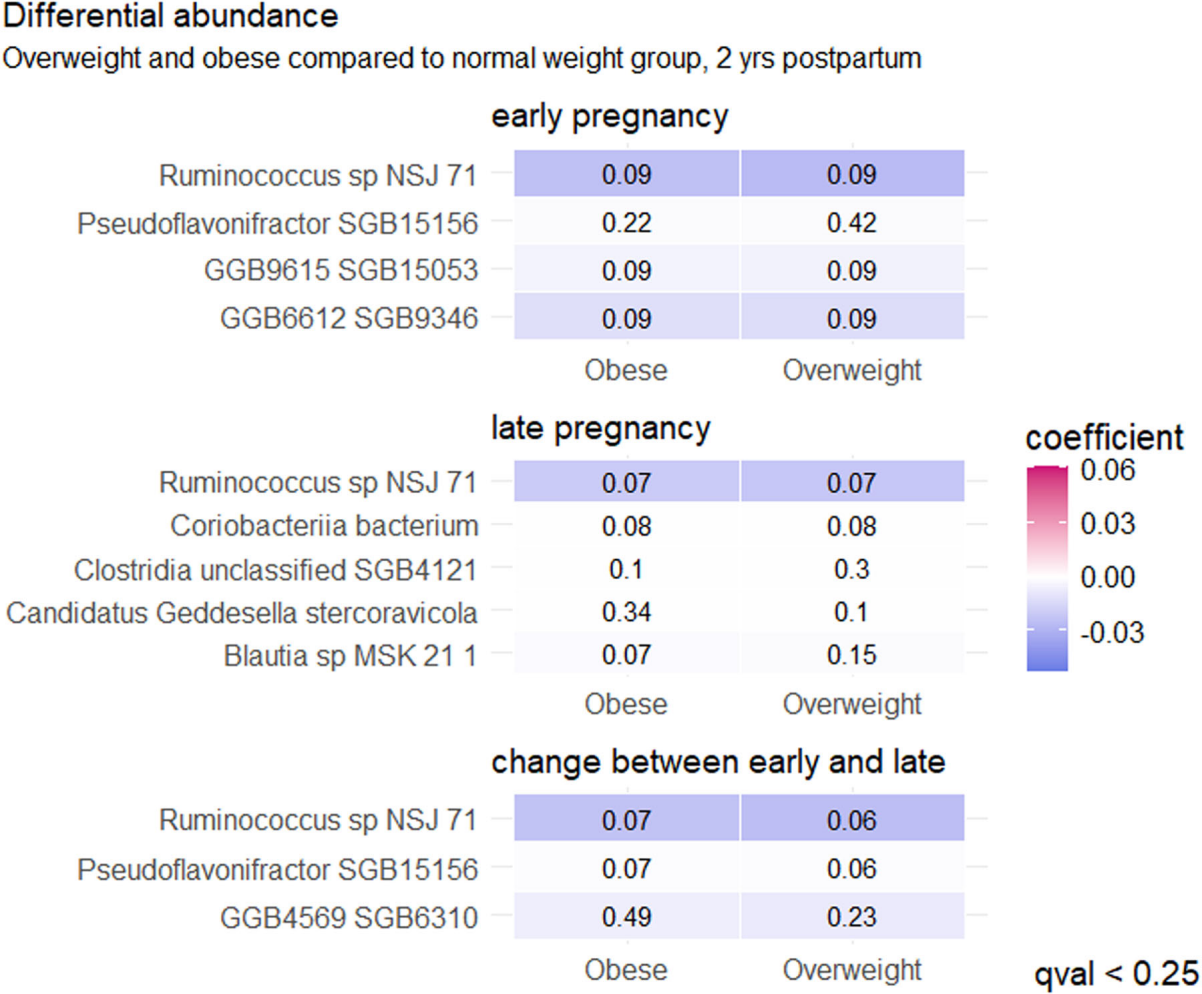
In early pregnancy, the relative abundances of *Ruminococcus* sp. NSJ 71, *Pseudoflavonifractor* SGB15156, GGB9615 SGB15053 (all belong to family *Ruminococcaceae*) and GGB6612 SGB9346 (family *Alphaproteobacteria* unclassified) were borderline significantly higher in women with normal weight as compared with those with overweight or obesity at 2‐years' postpartum (*q* < 0.25, MaAsLin2). In late pregnancy, the relative abundances of *Candidatus Geddesella stercoravicola*, *Clostridia* unclassified SGB4121 (members of family *Clostridia* unclassified), *Coriobacteriia bacterium* (family *Coriobacteriia* unclassified), and *Blautia* sp. MSK 21 1 (family *Lachnospiraceae*) were borderline significantly higher in women with normal‐weight women as compared with those with overweight and/or obesity (*q* < 0.25, MaAsLin2). The relative abundance of *Ruminococcus* sp. NSJ 71 increased both in women with obesity and overweight, while *Pseudoflavonifractor* SGB15156 increased only in women with obesity, but decreased in women with overweight, and GGB4569 SGB6310 (a member of family *Clostridia* unclassified) decreased in women with overweight (all borderline significant, *q* < 0.25, MaAsLin2). The color indicates the degree of the coefficient. In the heat map, the statistically significant findings are depicted with the FDR‐adjusted *p* value, that is, a *q* < 0.05 and the borderline statistically significant findings with a *q* < 0.25. FDR, False Discovery Rate.

**Figure 5:**
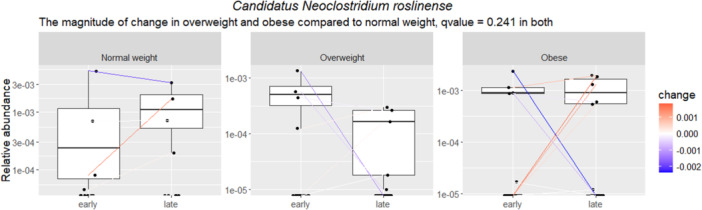
The relative abundance *Candidatus Neoclostridium roslinense* (a member of family *Clostridia* unclassified) decreased borderline significantly more in women with obesity and overweight as compared with women with normal weight at 5−6 years' postpartum (*q* = 0.24 for both, MaAsLin2). The color indicates the degree of change. The statistically significant findings are depicted with an FDR‐adjusted *p* value, that is, a *q* < 0.05 and the borderline statistically significant findings with a *q* < 0.25. FDR, False Discovery Rate.

It was found that the adiposity markers associated either statistically significantly (*q* < 0.05) or borderline significantly (*q* < 0.25) with relative abundance of bacterial species at early as well as in late pregnancy and with the change from early to late pregnancy (Tables S9–S11), mostly with BMI at 2‐years' postpartum (Table S9). In terms of bacterial species and BMI at 1‐year postpartum (Table S9), the relative abundance of GGB3034 SGB4030 (family *Erysipelotrichaceae*) in late pregnancy was higher in women with normal weight as compared with women with obesity (*q* = 0.02) and with overweight (*q* = 0.13) (Figure 3). At 2‐years' postpartum, eight bacterial species were borderline statistically significantly higher in early (*q* < 0.22) and late pregnancy (*q* < 0.15) in women with normal weight as compared with those in the overweight or obesity categories (Figure 4): higher relative abundances of *Ruminococcus* sp. NSJ 71, *Pseudoflavonifractor* SGB15156, GGB9615 SGB15053 (all belong to family *Ruminococcaceae*), *Candidatus Geddesella stercoravicola*, *Clostridia* unclassified SGB4121 (members of family *Clostridia* unclassified), GGB6612 SGB9346 (family *Alphaproteobacteria* unclassified), *Coriobacteriia bacterium* (family *Coriobacteriia* unclassified), and *Blautia* sp. MSK 21 1 (family *Lachnospiraceae*). At 2‐years' postpartum (Figure 4 and Table S9), borderline statistically significant changes during pregnancy in the relative abundance of three species during pregnancy were detected: *Ruminococcus* sp. NSJ 71 increased both in women with obesity (*q* = 0.06) and overweight (*q* = 0.07), while *Pseudoflavonifractor* SGB15156 increased only in women with obesity (*q* = 0.07), but decreased in women with overweight (*q* = 0.06). Furthermore, the abundances of GGB4569 SGB6310 (a member of family *Clostridia* unclassified) decreased in women with overweight (*q* = 0.23) (Figure 4). The only link between bacterial abundance and maternal adiposity measures at 5–6‐years' postpartum was a change in *Ca. Neoclostridium roslinense* (a member of the family *Clostridia* unclassified) during pregnancy; its relative abundance decreased borderline significantly more in women with obesity and overweight as compared with women with normal weight (*q* = 0.24 for both, Figure 5). No significant associations between BMI and species relative abundances during pregnancy were detected when BMI was analyzed as a continuous variable at any postpartum time points.

**Figure 6:**
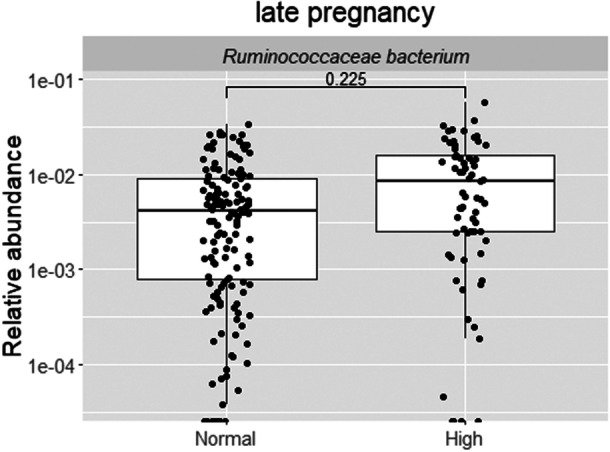
The relative abundance of *Ruminococcaceae bacterium* was borderline significantly higher in late pregnancy in women with higher WHR than in women with normal WHR at 1‐year postpartum (*q* = 0.23, MaAsLin2). The statistically significant findings are depicted with an FDR‐adjusted *p* value, that is, a *q* < 0.05 and the borderline statistically significant findings with a *q* < 0.25. FDR, False Discovery Rate; WHR, waist‐to‐hip ratio.

In terms of bacterial species and WHR, one borderline statistically significant finding was observed; the relative abundance of *Ruminococcaceae bacterium* was higher (borderline statistically significant, *q* = 0.23) in late pregnancy in women with higher (> 85 cm) WHR than women with normal WHR at 1‐year postpartum (Figure 6 and Table S10). Similarly, one borderline statistically significant observation was detected with fat%; a high fat% at 1‐year postpartum was associated with a lower abundance of GGB3034 SGB4030 in the late pregnancy (*q* = 0.17, Figure S5 and Table S11). There were no further associations detected between the values of WHR or body fat% or body fat% trajectories at any of the postpartum time points and the bacterial species present when the women were pregnant (Tables S10 and 11).

#### 3.2.4 Predicted Pathways

MetaCyc pathways linked to adiposity measures were predicted from the metagenomics profiles. Most of the relationships were detected between body fat% and late pregnancy pathways and only a few with early pregnancy pathways, which all were borderline statistically significant. With respect to BMI, early pregnancy dTDP‐α‐d‐ravidosamine and dTDP‐4‐acetyl‐α‐d‐ravidosamine biosynthesis (PWY_7688) was (*q* = 0.12) higher in women with normal weight as compared with overweight at 5–6‐years' postpartum (Figure S6 and Table S12). In terms of WHR, in late pregnancy, the level of chorismate biosynthesis from 3‐dehydroquinate (PWY‐6163) was higher in women with increased WHR than in women with normal WHR (*q* = 0.12) at 1‐year postpartum (Figure S7a and Table S13) and early pregnancy γ‐glutamyl cycle (PWY_4041) was lower in women with increased WHR when compared with those with normal WHR at 2‐years' postpartum (*q* = 0.20) (Figure S7b and Table S13). There were no further associations between BMI or WHR at any of the postpartum timepoints and predicted pathways observed during pregnancy.

Most of the findings were related to the values of body fat%, and with late pregnancy predicted pathways. At 1‐year postpartum, a high body fat% associated with low levels of glycogen biosynthesis I (from ADP‐d‐glucose) (*q* = 0.22) and sucrose biosynthesis II (PWY_7238) (*q* = 0.22). Furthermore, a high body fat% associated with high levels of phosphopantothenate biosynthesis I (*q* = 0.22), 6‐hydroxymethyl‐dihydropterin diphosphate biosynthesis I (PWY_6147), preQ. 0 biosynthesis (PWY‐6703) (*q* = 0.22), and thiamine diphosphate salvage II (*q* = 0.22) (Figure S8 and Table S14). The body fat trajectories were associated with four of these pathways: the levels of glycogen biosynthesis I (*q* = 0.13) and sucrose biosynthesis II (*q* = 0.17) were higher, while those of 6‐hydroxymethyl‐dihydropterin diphosphate biosynthesis I (PWY_6147) (*q* = 0.16) and preQ. 0 biosynthesis (*q* = 0.24) were lower in the decreasing and slowly rising group as compared with the women in the high and stable group (Figure S9 and Table S14). Furthermore, in comparison to the individuals in the high and stable group, the levels of l‐arginine biosynthesis II (acetyl cycle) (*q* = 0.24), mixed acid fermentation (*q* = 0.17), and glycogen degradation II (PWY_5941) (*q* = 0.24) were higher in the decreasing and slowly rising group (Figure S9 and Table S14). No other significant findings related to body fat% associations with early pregnancy or changes in predicted pathways were detected.

### 3.3 Prediction of Adiposity With the Composition of the Gut Microbiota and Predicted Functional Pathways During Pregnancy

The relative abundance of bacterial species and predicted functional pathways were observed to exhibit statistically significant predictive abilities with several adiposity markers at the 1‐year postpartum time point (Tables S15 and S16). Namely, the late pregnancy microbiota on its own and when combined with covariates and the change from early to late microbiota when combined with covariates, predicted BMI in the leave‐out test set at 1‐year postpartum, that is, the Random Forest model demonstrated significantly higher predictive performance compared with random baseline in late pregnancy (*p* < 0.001) and in the change between early and late pregnancy (*p* = 0.007), as evaluated using the AUROC value (Figure S10). Furthermore, the early pregnancy gut microbiota on its own and when combined with covariates and the change in gut microbiota on its own and when combined with covariates predicted the body fat% trajectories, that is, the Random Forest model demonstrated significantly higher predictive performance compared with random baseline in early pregnancy (*p* = 0.02) and in the change from early to late pregnancy (*p* < 0.001, Figure S11). Pregnancy gut microbiota was not found to predict WHR or body fat% during pregnancy or at any of the postpartum time points.

The species whose abundances were predictive of BMI at 1‐year postpartum are reported in Figure S12a–c. The following species that changed from early to late pregnancy were included as the most important features: bacterial species *Thomasclavelia spiroformis* (formerly *Clostridium spiroforme*, Lawson et al. 2023), *Ca. Allochristensenella caecavium*, *Veillonella dispar*, and GGB3005 SGB3996. The important bacterial species in late pregnancy were *Evtepia gabavorous*, *Enterocloster lavalensis*, *Agathobaculum butyriciproducens*, and *Lachnospira* SGB5076. The species predicting the body fat% trajectories are presented in Figure S13a–d. The most important features detected in the early pregnancy bacterial species *Ca. Allochristensenella caecavium*, GGB3571 SGB4778, *Lachnospira* SGB5076, *Clostridium* sp. AT4, GGB9615 SGB15053 and the change from early to late pregnancy were related to the following bacterial species; *Monoglobus pectinilyticus*, GGB9819 SGB15459, *Bifidobacterium pseudocatenulatum*, and *Dysosmobacter welbionis*.

Regarding the predicted functions, the early pregnancy and the change in the predicted functions predicted maternal BMI at 1‐year postpartum, that is, the Random Forest model demonstrated significantly higher predictive performance compared with random baseline in early pregnancy (*p* = 0.02) and in the change between early and late pregnancy (*p* < 0.001), as evaluated using AUROC value (Figure S14). Furthermore, there were functions in late pregnancy that predicted the body fat% trajectories when combined with covariates, that is, the Random Forest model demonstrated significantly higher predictive performance compared with a random baseline in late pregnancy (*p* = 0.01, Figure S15). Body fat%, when assessed as a continuous variable at 1‐year postpartum, was predicted by the change in the pathways (Figure S16). The predicted functions did not predict WHR at any postpartum time point.

The functions which predicted the BMI at 1‐year postpartum are reported in Figure S17a–c. The most important features included the following metabolic systems: the early pregnancy tricarboxylic acid cycle (TCA) cycle I (prokaryotic), l‐lysine biosynthesis VI, cytidine disphosphate (CDP)‐diacylglycerol biosynthesis I, superpathway of *N*‐acetylglucosamine, *N*‐acetylmannosamine, and *N*‐acetylneuraminate degradation, superpathway of coenzyme A biosynthesis and the change from early to late pregnancy of TCA cycle VII (acetate‐producers), superpathway of pyrimidine ribonucleosides degradation, hexitol fermentation to lactate, formate, ethanol, and acetate, the superpathway of glyoxylate cycle, fatty acid degradation, and pentose phosphate pathway (nonoxidative branch) I. The pathways predicting the body fat% trajectories are reported in Figure S18. The most important features with regard to prediction were dTDP‐β‐l‐rhamnose biosynthesis, l‐lysine biosynthesis I, mixed acid fermentation, and the superpathway of branched chain amino acid biosynthesis. Pathways predicting body fat% at 1‐year postpartum are reported in Figure S19a,b. The Random Forest model demonstrated significantly higher performance compared with a random baseline in the change between early and late pregnancy (*p* = 0.0052, Figure S16). In this respect, the most important features were 9‐*cis*, 11‐*trans*‐octadecadienoyl‐CoA degradation (isomerase‐dependent, yeast), UDP‐*N*‐acetyl‐d‐glucosamine biosynthesis, 6‐gingerol analog biosynthesis (engineered), and the superpathway of menaquinol‐6 biosynthesis.

## 4 Discussion

Our findings show that the composition and function of the gut microbiota during pregnancy are linked to postpartum maternal adiposity. Particularly, the community diversity of the gut microbiota (*α*‐diversity) was related to body fat% at 1‐year postpartum, while the relative abundances of specific bacterial species associated mostly with BMI at 2‐years' postpartum.

We showed that community diversity was associated with the body fat%. The observed effects in this study were small, but statistically significant, for example, each single unit increase in body fat% was associated with a 0.1 decrease in Shannon diversity. In general, the determination of body fat% requires more resources than the traditionally used assessment of BMI, but it provides a compositional view of adiposity. To our knowledge, there are only a few studies that have probed the relationship between body fat% and the gut microbiota as analyzed by metagenomics, and the existing investigations have been conducted in nonpregnant subjects (Le Roy et al. 2019; Petersen et al. 2021). These previous studies have linked differential abundances in the gut microbiota with body fat% but not with diversity, and thus our finding can be considered novel. In contrast, community diversity was not associated with BMI or WHR at any postpartum time point. In general, according to the systematic review published by Pinart et al. (2021), a lower community diversity has been reported in obese versus nonobese adults as defined by BMI, but the results have been inconclusive.

In addition to diversity, we found distinct relative abundances of specific bacteria according to maternal adiposity status at all three postpartum time points. These findings, except one, were all borderline statistically significant (*q* = 0.06–0.24), indicating that it might be possible that with a higher number of subjects, these could reach statistical significance. The difference in the relative abundance of GGB3034 SGB4030 (family *Erysipelotrichaceae*) in late pregnancy between women with normal weight and women with obesity was statistically significant (*q* = 0.02). It is noteworthy that a low abundance of this same bacterial species was associated with a high body fat% at 1‐year postpartum; however, this finding was borderline statistically significant. There are no other studies that would have investigated the relationship of gut microbiota during pregnancy to postpartum adiposity. However, those publications that have focused on gut microbiota and adiposity during pregnancy have reported heterogeneous findings (Houttu et al. 2018; Ruebel et al. 2021). Nonetheless, in these previous studies, 16S ribosomal RNA (rRNA) sequencing, a less accurate method compared with metagenomics sequencing, was applied. Furthermore, the findings were reported using less robust significance levels and/or only at the genus level, as is appropriate for 16S analytics. One recent report did apply a metagenomics approach (Dreisbach et al. 2023) and found differences in composition and predicted function during pregnancy according to the pregravid BMI value. Our study is the first to investigate the contribution of gut microbiota during pregnancy to maternal adiposity measured as long as 6 years after delivery.

The strongest evidence of the association between species abundance in pregnant women was related to BMI at 2‐years' postpartum: A total of 9 species were linked to BMI, while only one species was related to BMI at 1‐year postpartum and one at 5–6‐years' postpartum. With respect to these species, we consider that *Ruminococcus* sp. NSJ 71 and *Pseudoflavonifractor* SGB15156 demonstrated the most interesting relationships, as both exhibited distinct changes during pregnancy as well as a higher abundance in both early and late pregnancy in women who were normal weight at 2‐years' postpartum. These species are members of the family of *Ruminococcaceae* and both higher and lower relative abundances of this family have been related to GDM (Mokkala et al. 2017; Wang et al. 2020). Furthermore, lower abundances of other members of *Ruminococcaceae* family during pregnancy have been detected in women with incident prediabetes at 2‐years' postpartum (Houttu et al. 2023). These heterogeneous findings may originate from the different metabolic capabilities of species in the *Ruminoccaceae* family, for example, their abilities to produce short‐chain fatty acids (SCFAs) (P. Louis and Flint 2017). In one previous study, *Pseudoflavonifractor* was enriched at baseline in subjects with obesity who lost weight as compared with those who were less successful in this respect (S. Louis et al. 2016). There is one report of a decrease in the abundance of *Pseudoflavonifractor* spp. in prediabetes and in type 2 diabetes subjects when compared with low‐risk (for type 2 diabetes) normal glucose tolerance subjects (Wu et al. 2020). Although the current knowledge of the specific bacterial species is still in its infancy, the previous studies have hinted at an association between *Pseudoflavonifactor, Ruminoccaceae* and metabolic complications, suggesting that there may be a link between these bacteria and metabolic health.

The relations of BMI with other species were observed only at a certain stage of the pregnancy in women with normal weight, for example, higher abundances were found mostly in species of the class *Clostridia*. Class *Clostridia* is known to include bacteria capable of polysaccharide fermentation, and thus these species are potential SCFA producers. Similarly, in women of normal weight, a higher abundance of species in the *Blautia* genus was detected in pregnant women. In general, the presence of *Blautia* in the human gut has been associated with both positive and negative features (Maturana and Cárdenas 2021); these microbes are known to be carbohydrate‐fermenting bacteria and thus also to be potential SCFA producers. Furthermore, different abundances and changes during pregnancy were observed in several less well‐characterized species (GGBs/SGBs in the family *Alphaproteobacteria* and *Erysipelotrichaceae*, as well as in the class *Clostridia*, species *C. bacterium* [genus and family *Coriobacteriia* unclassified] and *Ca. Neoclostridium roslinense* [unclassified family of class *Clostridia*]).

Regarding the functional potential of the gut microbiota, most of the associations with maternal adiposity markers were detected in late pregnancy functions and body fat% at 1‐year postpartum. Interestingly, the glycogen biosynthesis I (from ADP‐d‐glucose) pathway, which was associated with a high body fat%, has been related to an adaptation to low carbohydrate availability in bacteria (Esteban‐Torres et al. 2023). Moreover, the sucrose biosynthesis II pathway is also the biosynthesis pathway from monosaccharides to more complex sugars that might promote an adaptation to a dietary stress such as starvation and mixed acid fermentation can also represent a means to digest more difficult substrates, and finally, glycogen degradation might be used by those types of bacteria that also have the capability for glycogen biosynthesis. This glycogen may be accessed when the glycogen stores are depleted during starvation. One association between functional pathways and BMI at 5–6‐years' postpartum was detected. It needs to be noted that all the findings related to functional pathways were borderline statistically significant. Moreover, some of the pathways are linked to molecules that are antibacterial, suggesting that they may exert protective roles against other potentially pathogenic bacteria. Overall, the pathways were mostly related to bacterial housekeeping and metabolic pathways and their clinical significance remains to be elucidated.

The strongest, but still rather weak prediction of adiposity, was detected between the change in gut microbiota from early to late pregnancy and the body fat% trajectories. In addition, predicted functions predicted BMI at 1‐year postpartum and, less impressively, with body fat% trajectories. One previous study investigating the potential of gut microbiota as analyzed by a metagenomics approach to predict adiposity applied only the value of the BMI in a nonpregnant population (Liang et al. 2023). We have additionally used body fat% as well as body fat% trajectories, which is undoubtedly a more reliable and modern way to assess the development of adiposity over time.

The strength of our study lies in the comprehensive data obtained utilizing three different adiposity measures. We measured body fat% by air displacement plethysmography, which is comparable to the gold standard technique for measuring body composition, that is, underwater weighing. It is acknowledged that body fat% is a better measure of adiposity than BMI. For this reason, we utilized the fat% measures in the body fat% trajectories; this is a novel way to assess the development of adiposity over a longer period, and this makes these results unique. Second, the comprehensive metadata permitted us to exclude users of antibiotic agents as well as antidiabetic drugs, metformin, and insulin, because those drugs can have a major effect on the gut microbiota. Third, the gut microbiota was analyzed using metagenomics, which reaches a finer level of taxonomy than the traditionally used 16S rRNA gene sequencing. Moreover, the study setting with its long‐term follow‐up allowed the investigation of the relationship between the gut microbiota present during pregnancy and the adiposity measures at three postpartum time‐points, an approach not attempted previously. The limitations include the lack of normal‐weight women at study entry. Subsequently, the sample sizes for women with normal weight at postpartum time‐points were small. In this prospective study setting, the sample sizes for groups in postpartum time‐points could not be chosen; thus, we call for further studies with larger sample sizes, including women of normal weight. Moreover, it should be noted that the microbial functional pathways were predicted, not measured.

Overweight and obesity during pregnancy predispose the mother to metabolic complications, such as GDM during pregnancy, and diabetes in later life. Maternal metabolism is a strong contributor both to GDM and the incidence of prediabetes (Mokkala et al. 2020; Muhli et al. 2023), which can lead to the development of type 2 diabetes. Whether the composition of the gut microbiota during pregnancy has a similar importance in these disorders has been less extensively studied nor is it recognized whether the gut microbiota present during pregnancy can influence the women's postpartum lives. Here, we demonstrate that the presence of specific gut microbiota species during pregnancy is related to long‐term postpartum adiposity. Thus, it may be possible to modify the risk for obesity and other metabolic complications by modifying the composition of the gut microbiota.

To conclude, we have detected a link between gut microbiota diversity and bacterial species in pregnant women with postpartum adiposity, with many of the relationships being detectable at 2‐years' postpartum. These findings highlight the mediating role of the gut microbiota during pregnancy in influencing the overweight and obesity condition postpartum. It is evident that more studies are needed to clarify the significance of the gut microbiota during pregnancy on the long‐term health of women.

## Author Contributions

**Noora Houttu:** conceptualization, investigation, visualization, writing – original draft, writing – review and editing. **Kati Mokkala:** investigation, writing – original draft, writing – review and editing. **Himmi Lindgren:** formal analysis, visualization, writing – review and editing. **Mrunalini Lotankar:** writing – review and editing. **Chouaib Benchraka:** formal analysis, writing – review and editing. **Katariina Pärnänen:** software, writing – review and editing. **Lotta Saros:** investigation, writing – review and editing. **Ella Muhli:** writing – review and editing. **Tero Vahlberg:** formal analysis, writing – review and editing. **Leo Lahti:** software, supervision, writing – review and editing. **Kirsi Laitinen:** conceptualization, supervision, funding acquisition, project administration, resources, writing – review and editing.

## Ethics Statement

The study complied with the Declaration of Helsinki 2023. The Ethics Committee of the Hospital District of Southwest Finland approved the study protocol, and women provided written informed consent.

## Conflicts of Interest

The authors declare no conflicts of interest.

## Supporting information

Supplemental tables 1–8.

Supplemental tables 9–11.

Supplemental tables 12–16.

Supplemental figures.

## Data Availability

The data sets are not available due to their containing information that could compromise participant privacy and consent, but are available from the corresponding author upon reasonable request and subject to a collaboration agreement. The source code for the analyses is available in Zenodo (https://doi.org/10.5281/zenodo.17548526).
