# Supplementary material for: The Relationship Between Gut Microbiota During Pregnancy and the Level of Postpartum Adiposity
Source: Microbiologyopen. 2025 Nov 12;14(6):e70128. doi: 10.1002/mbo3.70128 (PMC12612556; doi:10.1002/mbo3.70128)
Supplement: Supplementary file 4 — Supplemental figures. [file MBO3-14-e70128-s003.docx]

a)


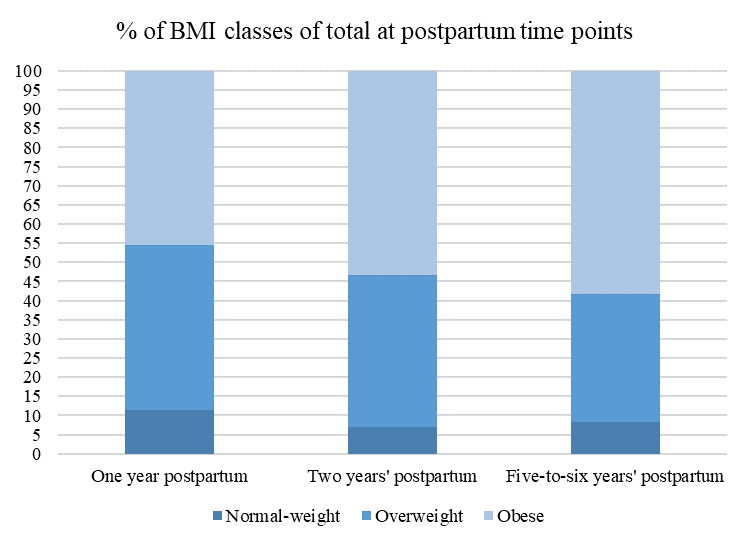


b)


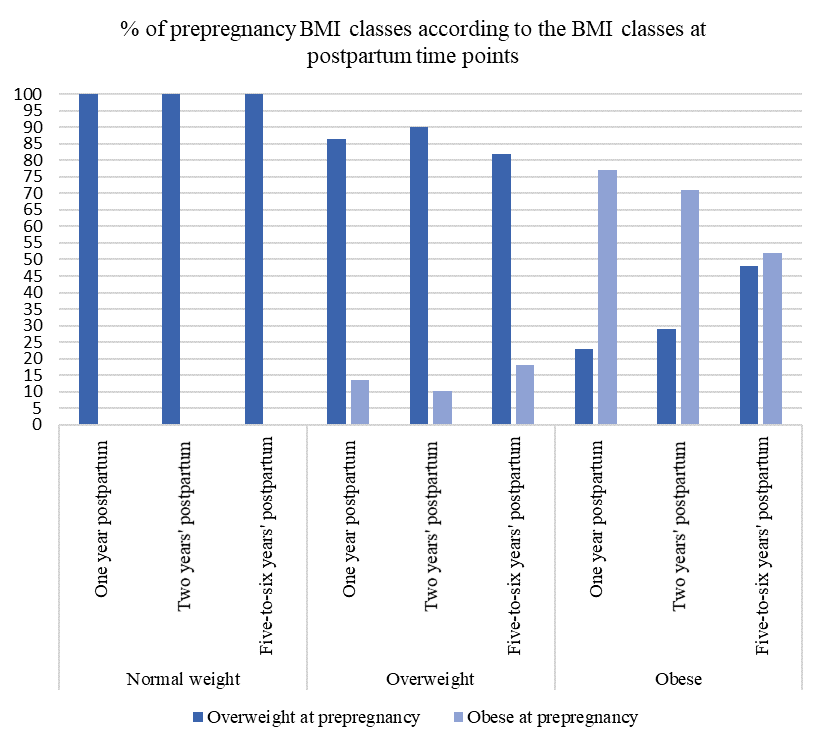


Supplemental Figure 1a-b. a) The % of women with normal weight, overweight and obesity at one, two and five-to-six years’ postpartum. b) The % of the women with normal weight, overweight and obesity based on prepregnancy BMI according to the postpartum groups of women with normal weight, overweight and obesity and postpartum time points.

a)


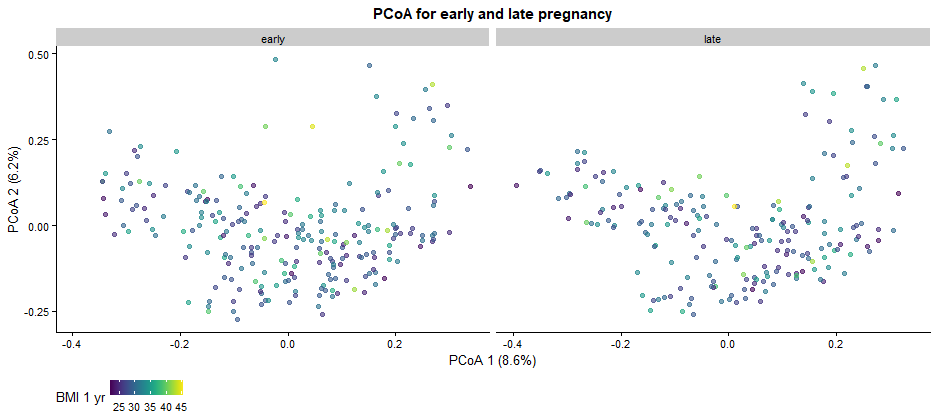


b)


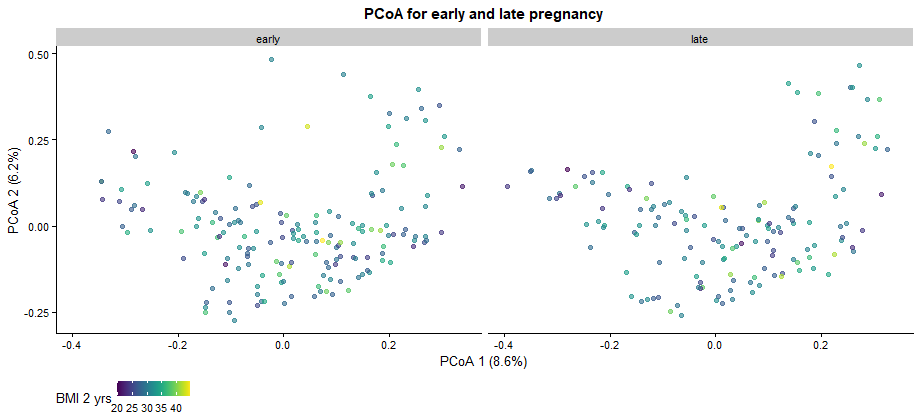
Supplemental Figure 2a-b. a) Principal Coordinate Analysis (PCoA) ordination revealing dissimilarities in the gut microbiota among the women in early and late pregnancy. The BMI at one year postpartum is indicated by color. b) Principal Coordinate Analysis (PCoA) ordination showing dissimilarities in the gut microbiota among the women in early and late pregnancy. The BMI at two years’ postpartum is indicated by color.


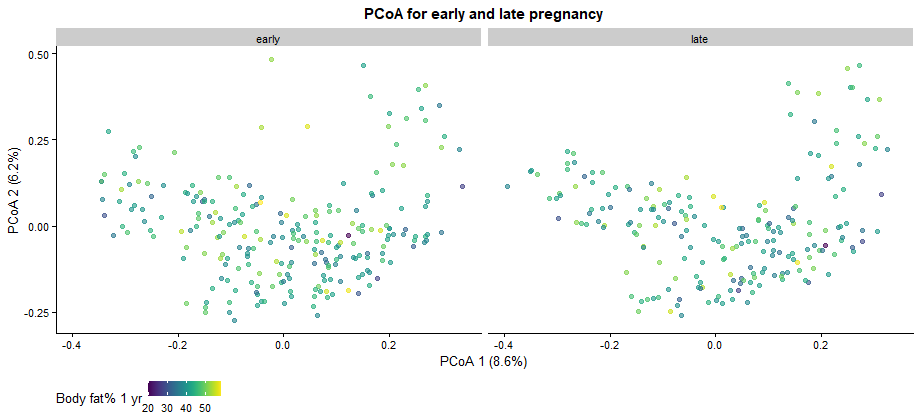


Supplemental Figure 3. Principal Coordinate Analysis (PCoA) ordination depicting dissimilarities in the gut microbiota among the women in early and late pregnancy. The body fat% at one year postpartum is indicated by color.


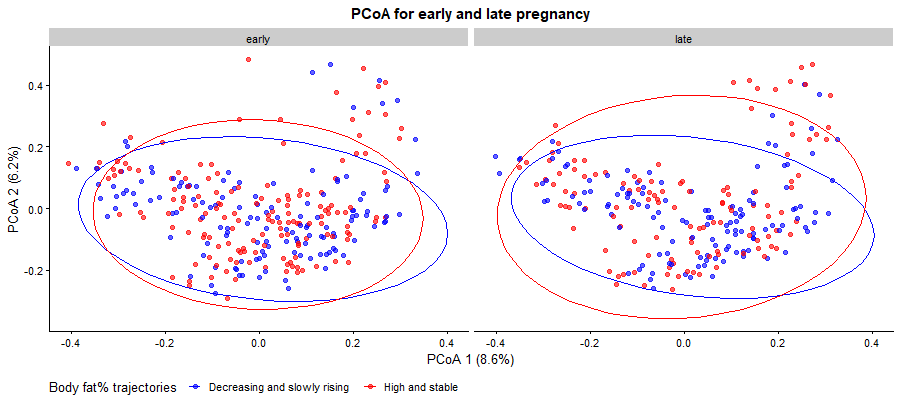


Supplemental Figure 4. Principal Coordinate Analysis (PCoA) ordination of gut microbiota dissimilarities among the women in early and late pregnancy. The body fat% trajectories at one year postpartum indicated by color.


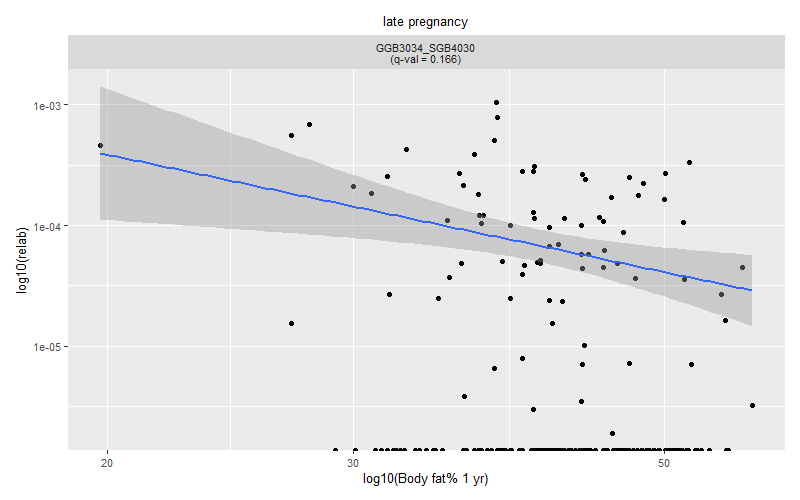


Supplemental Figure 5. The body fat% at one year postpartum associated with the relative abundance of GGB3034 SGB4030 (family *Erysipelotrichaceae*) in late pregnancy (q=0.17, MaAsLin2).


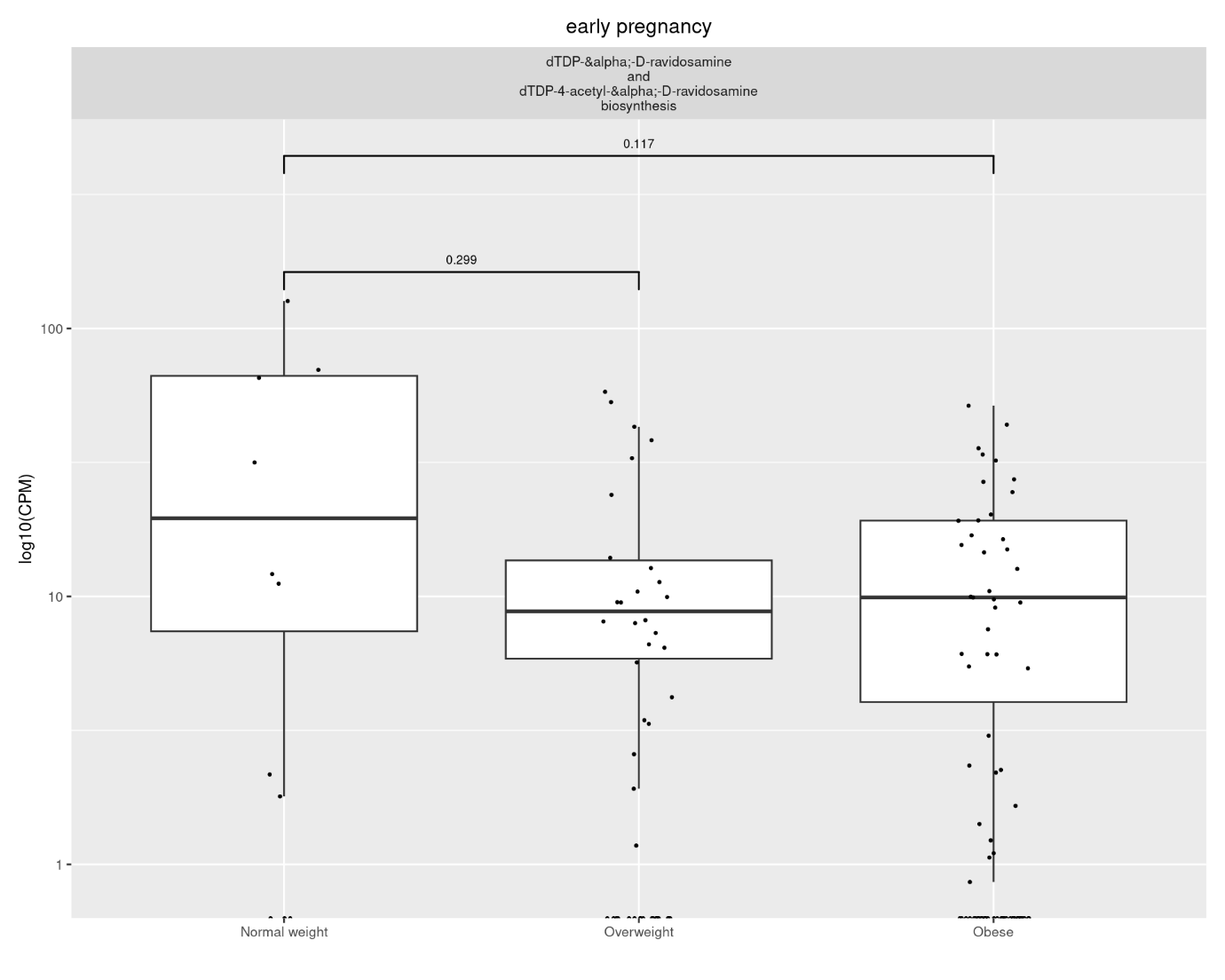


Supplemental Figure 6. The levels of dTDP-α-D-ravidosamine and dTDP-4-acetyl-α-D-ravidosamine biosynthesis in early pregnancy were higher in women with normal-weight as compared to those who were living with overweight and obesity at 5-6 years’ postpartum (q<0.30 for both, MaAsLin2).

a)


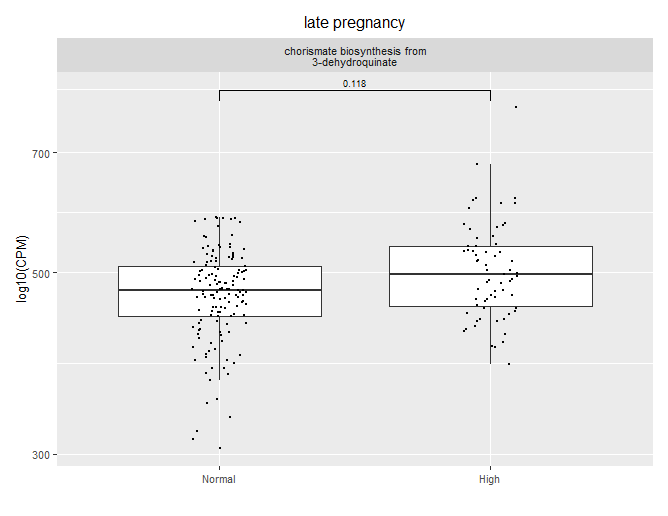


b)


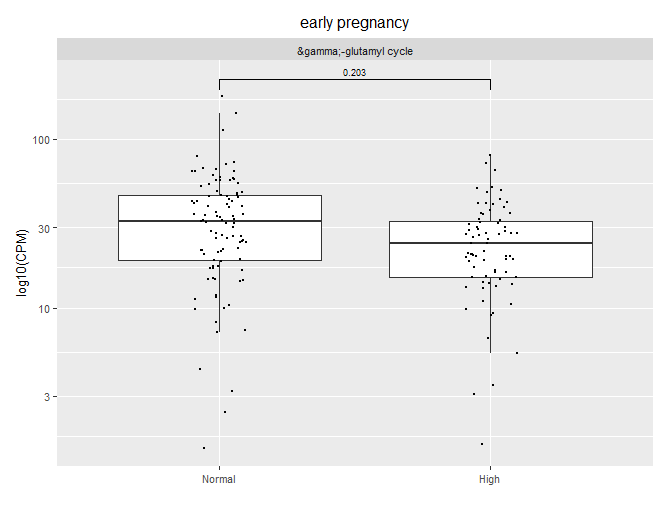


Supplemental Figure 7a-b. a) The level of chorismate biosynthesis from 3-dehydroquinate in late pregnancy was lower in women with normal WHR as compared to women with high WHR at one year postpartum (q=0.12, MaAsLin2). b) The γ-Glutamyl cycle in early pregnancy was higher in women with normal WHR as compared to women with high WHR at two years’ postpartum (q=0.20, MaAsLin2).


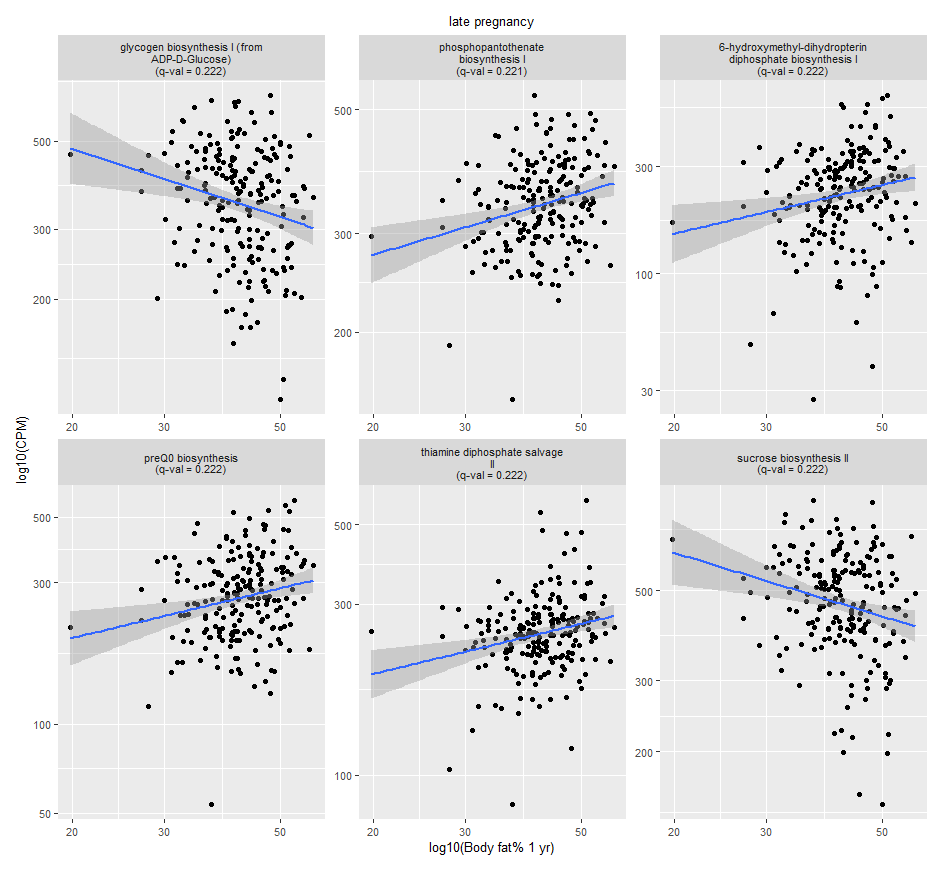


Supplemental Figure 8. The body fat% at one year postpartum associated directly or indirectly with functional pathways in late pregnancy (q<0.22 for all, MaAsLin2).


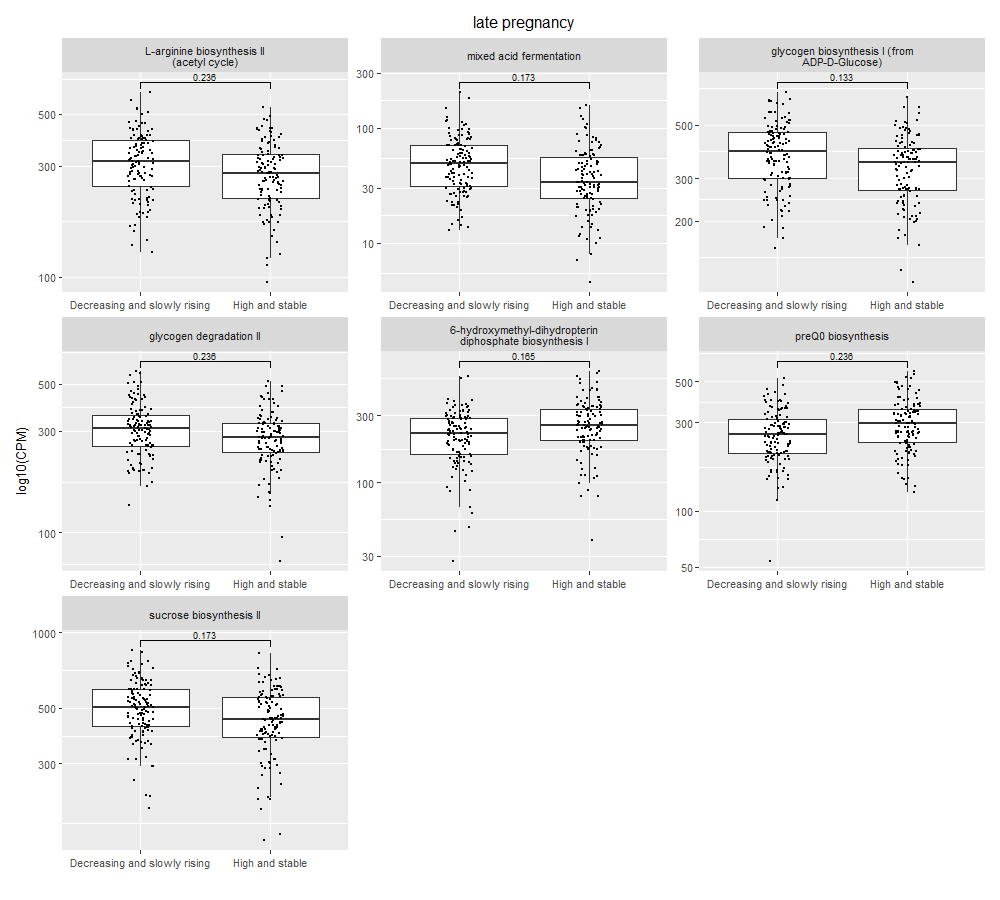


Supplemental Figure 9. The pathways in late pregnancy, which differed between women with 1) decreasing and slowly rising and 2) high and stable of body fat% trajectories (q<0.24 for all, MaAsLin2).


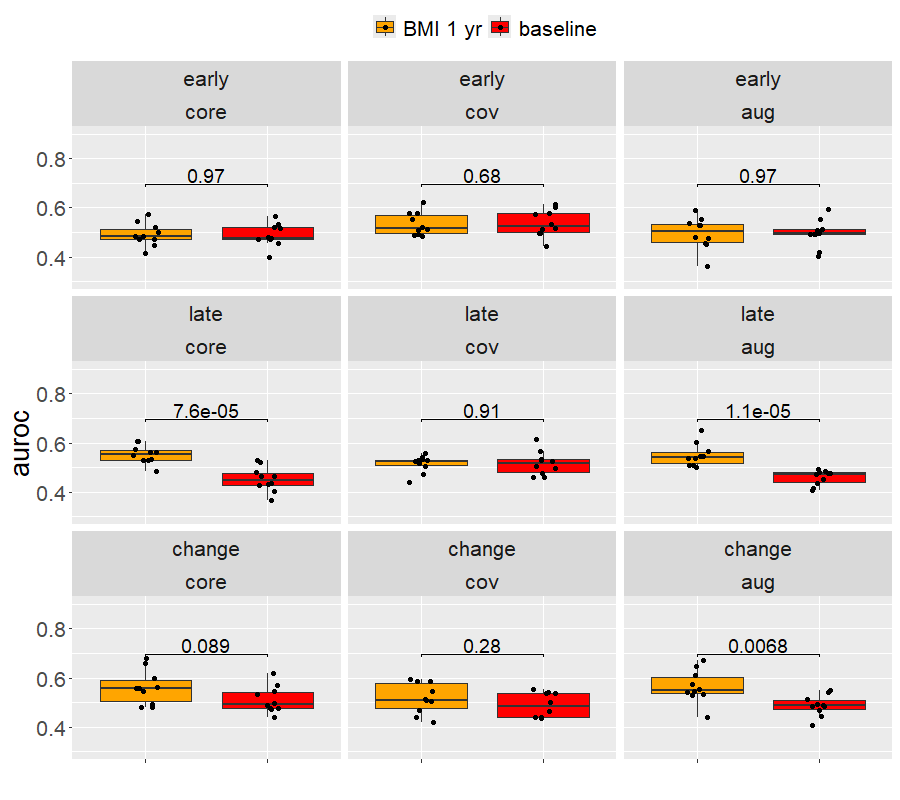
Supplemental Figure 10. The prediction performance of categorised BMI as quantified by AUROC at one year postpartum with gut microbiota in early and late pregnancy and with its change. Late pregnancy microbiota (p<0.001, Wilcoxon rank-sum test) and when combined with covariates (p<0.001, Wilcoxon rank-sum test) and the change from early to late microbiota when combined with covariates (p=0.007, Wilcoxon rank-sum test), predicted BMI in the leave-out test set at one year postpartum.


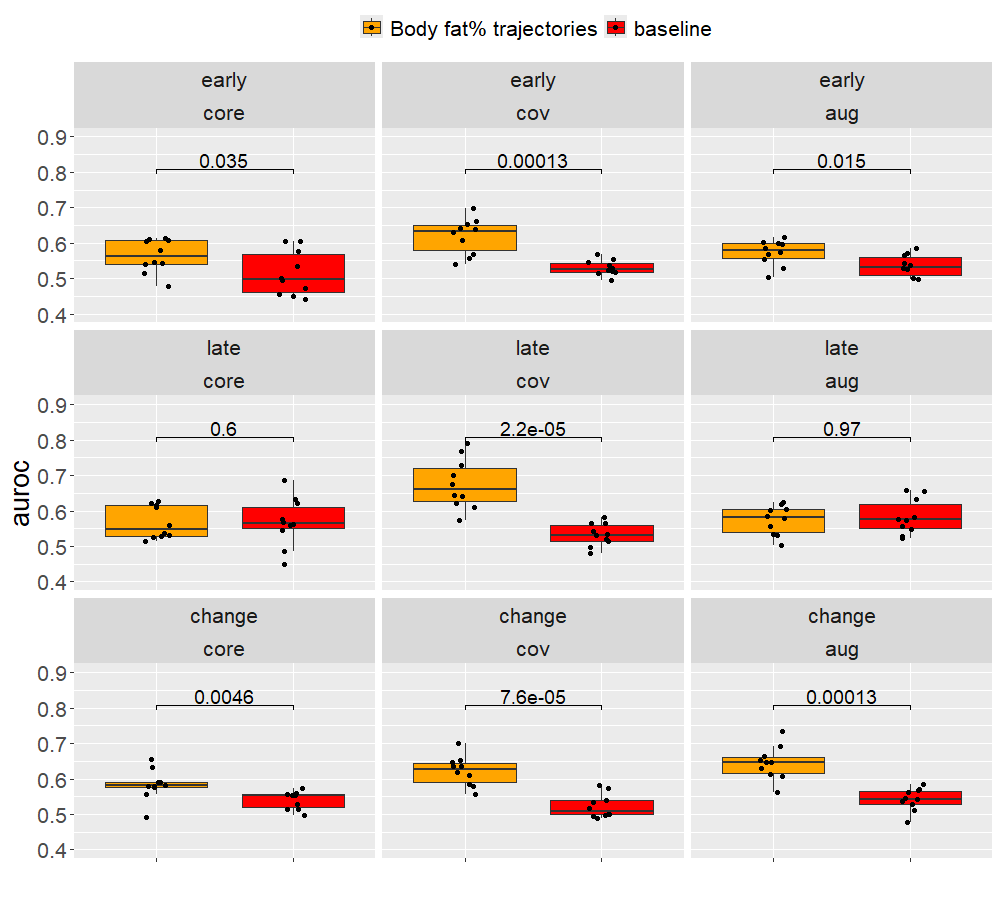
Supplemental Figure 11. The prediction performance of body fat% trajectories quantified by AUROC with gut microbiota in early and late pregnancy and with its change. Early pregnancy gut microbiota (p=0.04, Wilcoxon rank-sum test) and when combined with covariates (p=0.02, Wilcoxon rank-sum test) and the change in the gut microbiota (q=0.005, Wilcoxon rank-sum test) and when combined with covariates (p<0.001, Wilcoxon rank-sum test) predicted the body fat% trajectories

a)


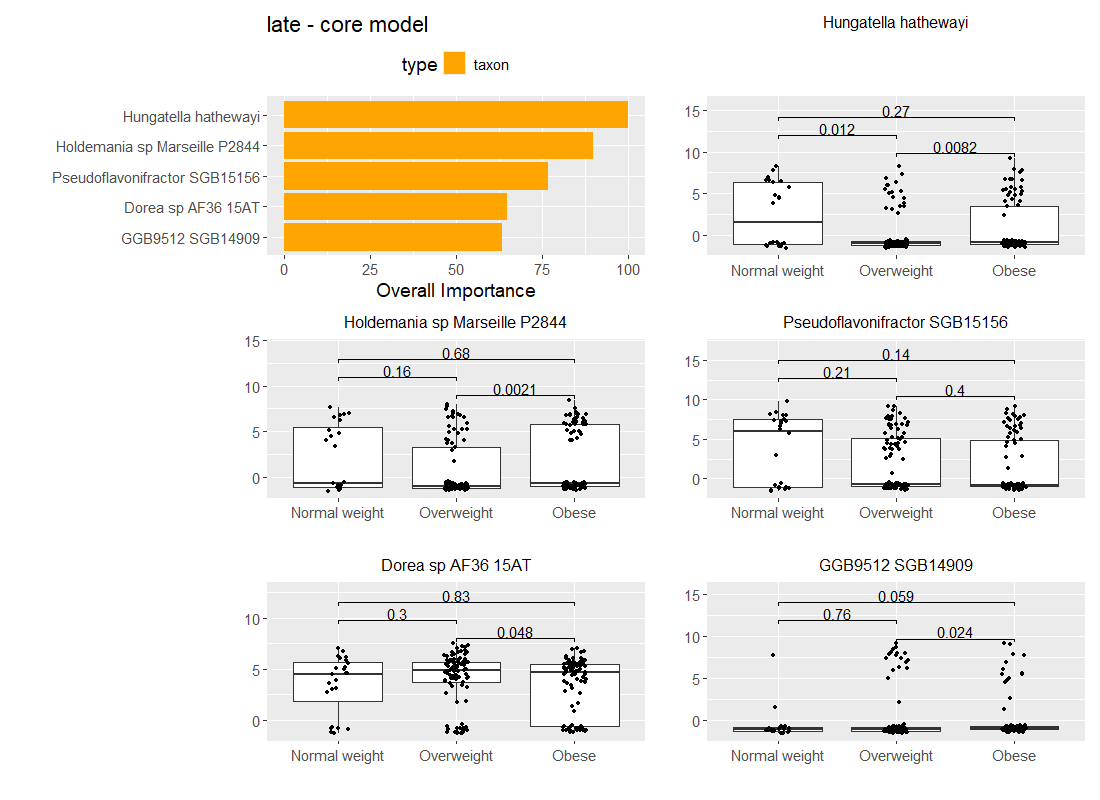


b)


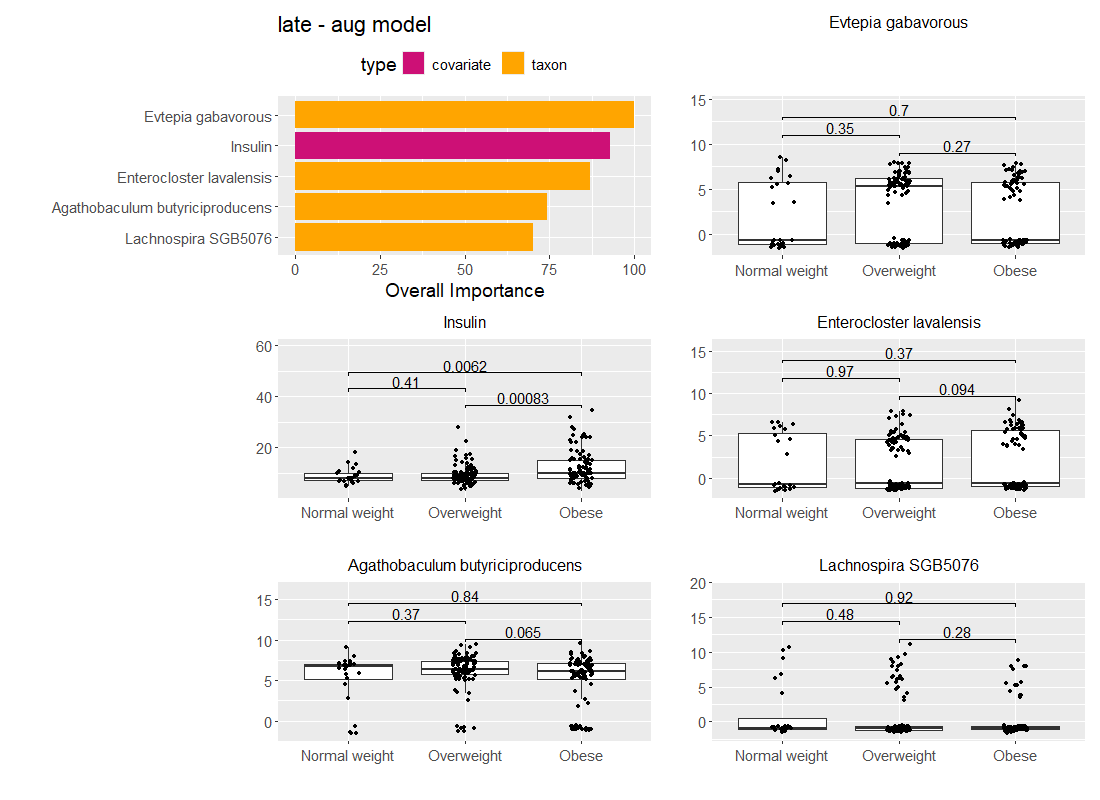


c)
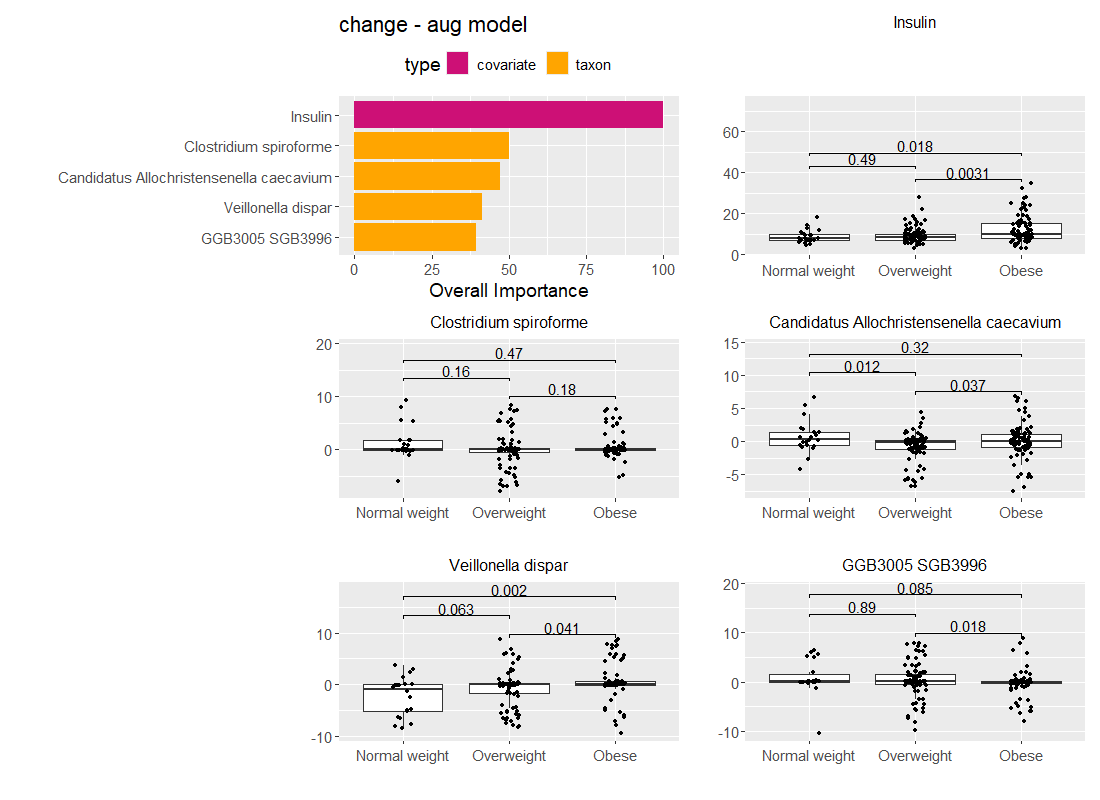


Supplemental Figure 12a-c. The presence of certain bacteria in late pregnancy (a-b) and the change from early to late pregnancy (c) that predict BMI at one year postpartum. Testing between the groups is done with Wilcoxon rank-sum test and the statistical difference is depicted with a p-value.

a)


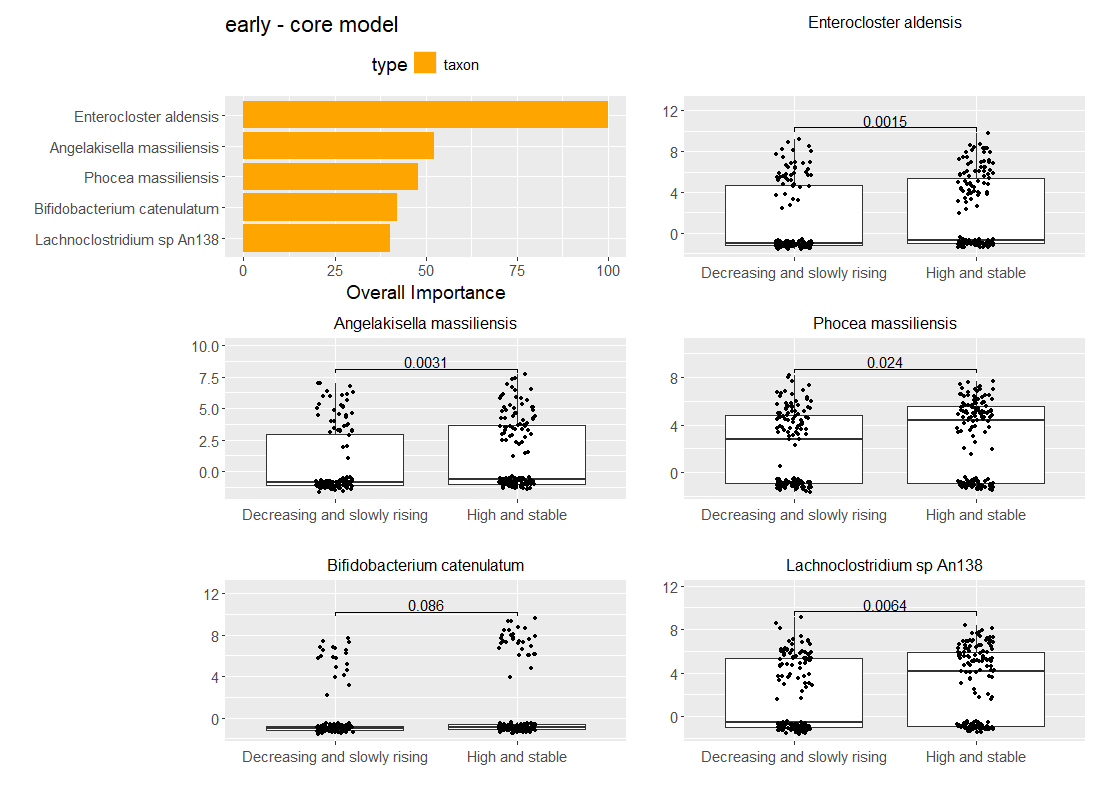


b)


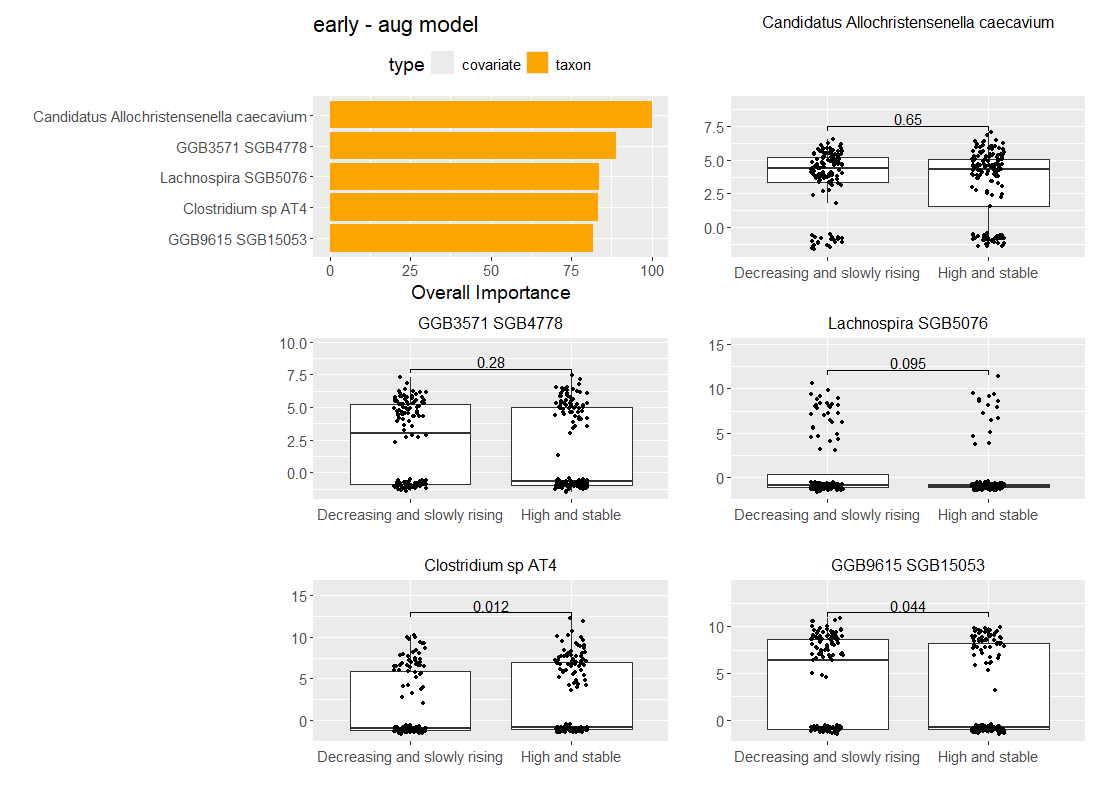


c)


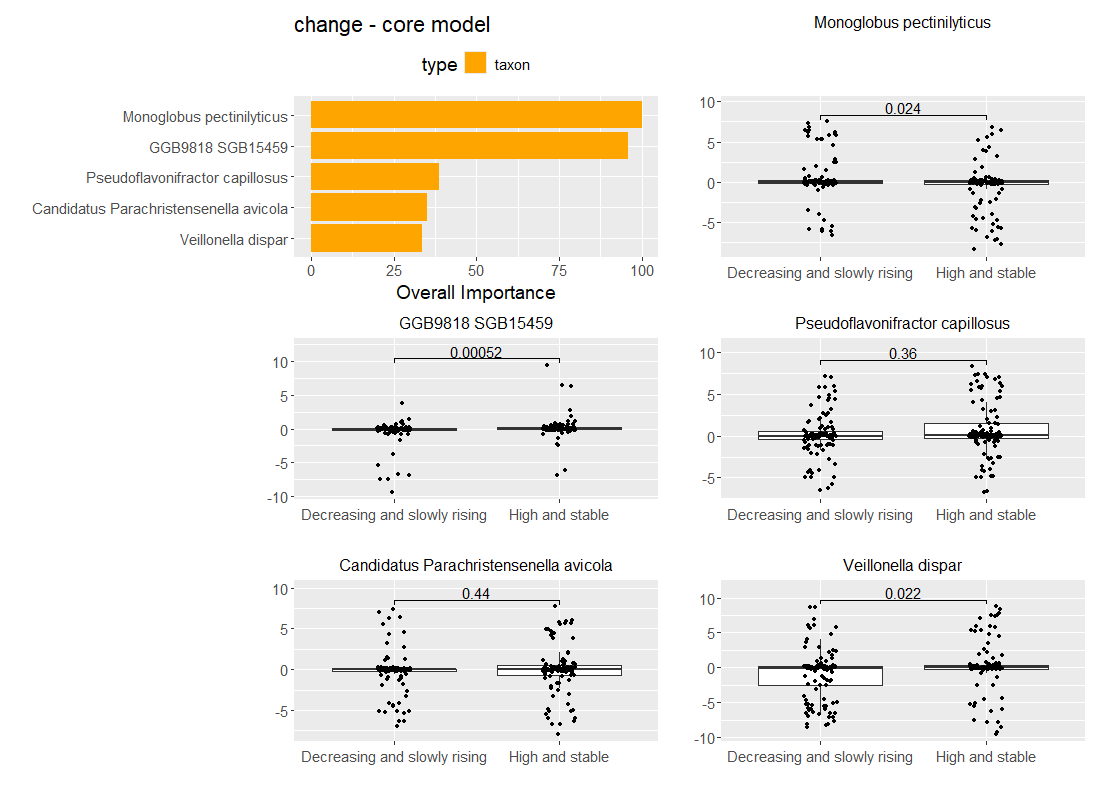


d)


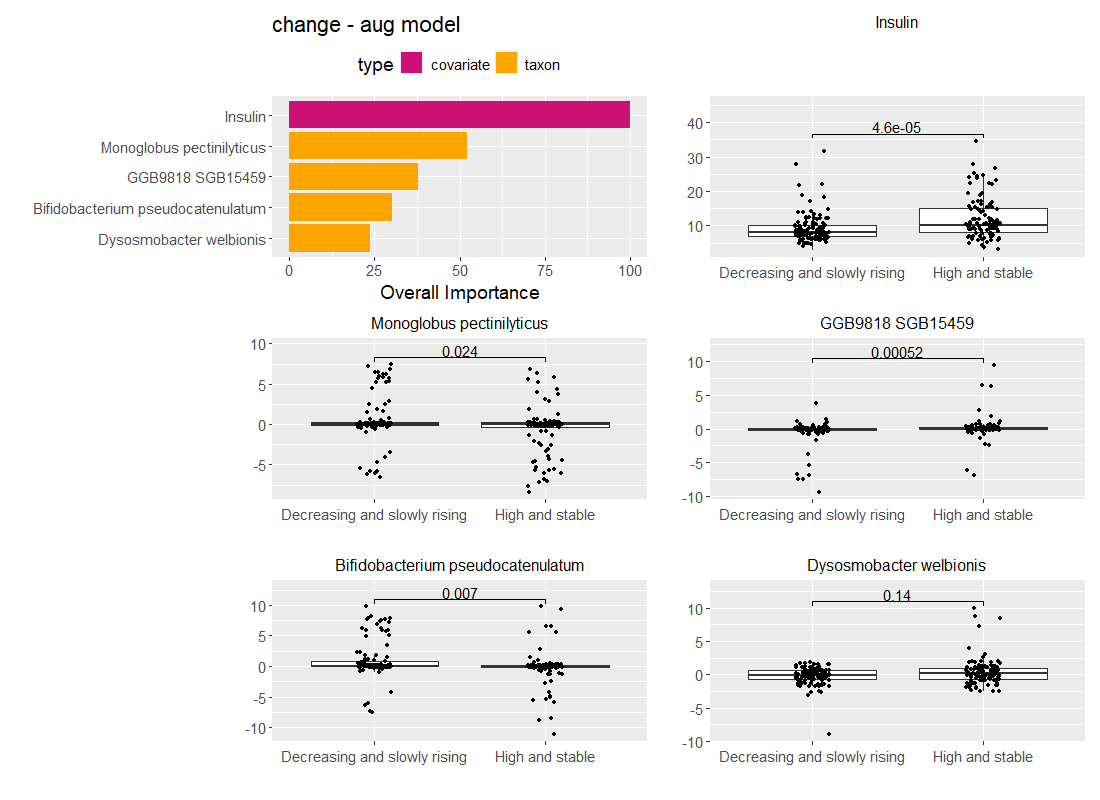


Supplemental Figure 13a-d. The presences of certain bacteria in early pregnancy (a-b) and the change from early to late pregnancy (c-d) predicting the body fat% trajectories. Testing between the groups is done with the Wilcoxon rank-sum test and the statistical difference is depicted with a p-value.


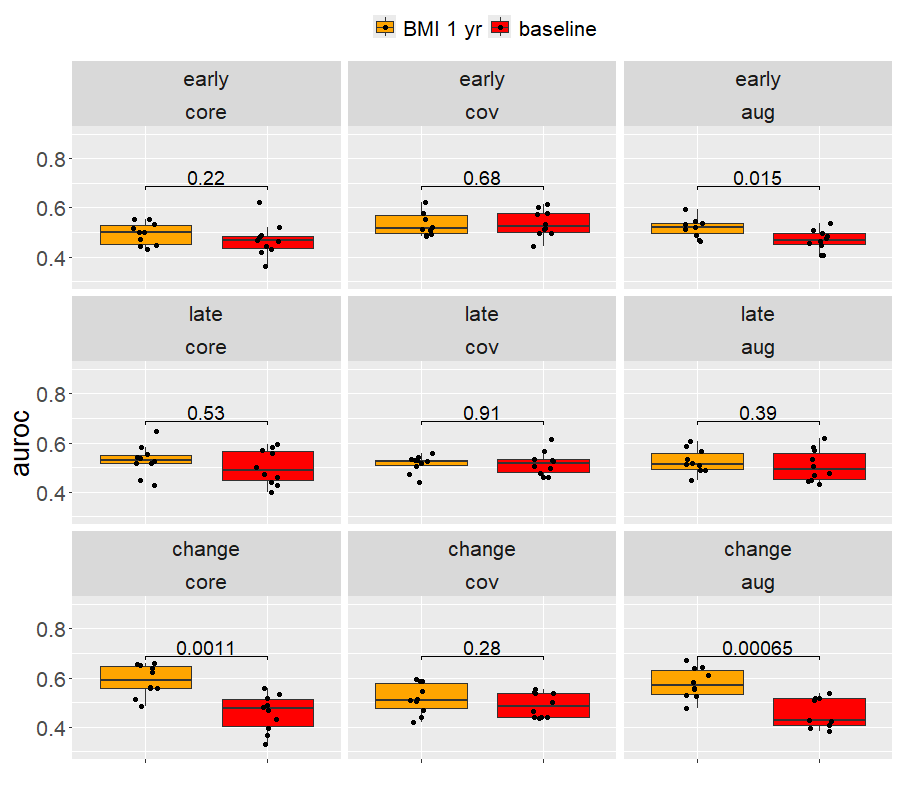
Supplemental Figure 14. The prediction performance of categorised BMI quantified by AUROC at one year postpartum with pathways in early and late pregnancy and with its change. Early microbiota when combined with covariates (p=0.02, Wilcoxon rank-sum test) and change in microbiota when combined with covariates (p<0.001, Wilcoxon rank-sum test) predicted BMI at one year postpartum.


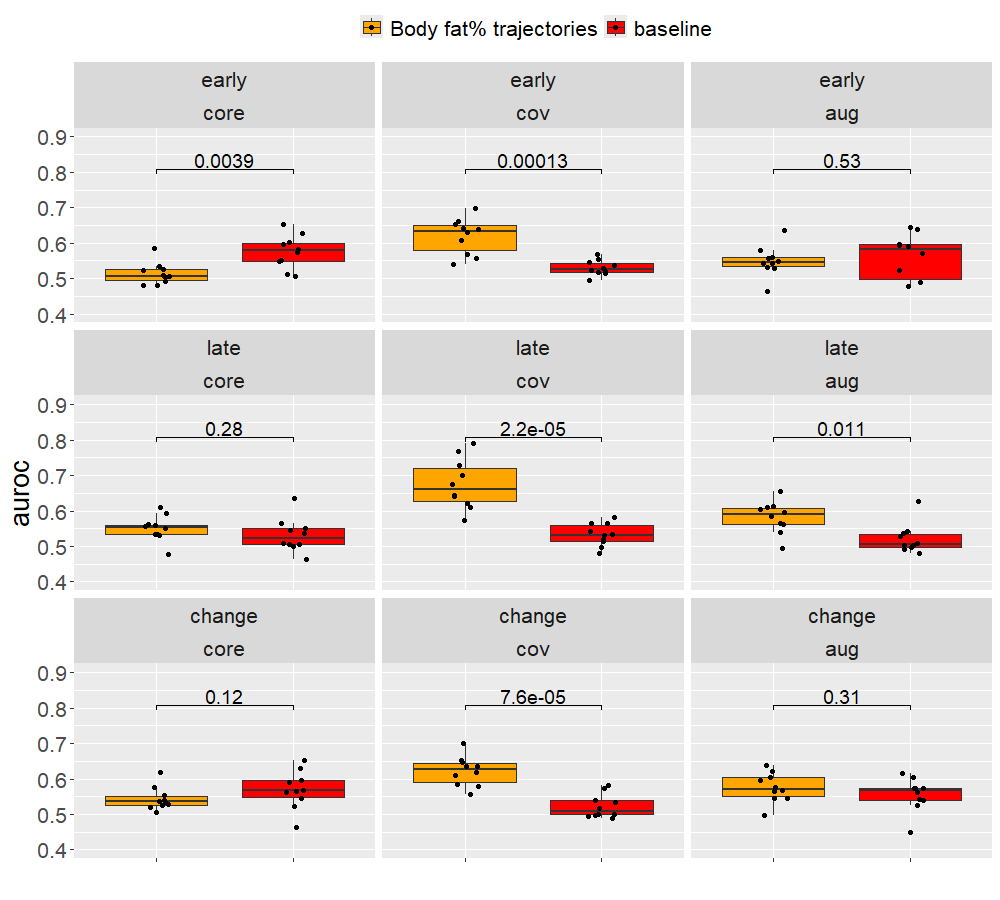
Supplemental Figure 15. The prediction performance of body fat% trajectories quantified by AUROC with pathways in early and late pregnancy and with its change. Early microbiota (p=0.004, Wilcoxon rank-sum test) and late microbiota combined with covariates (p=0.01, Wilcoxon rank-sum test) predicted body fat% trajectories.


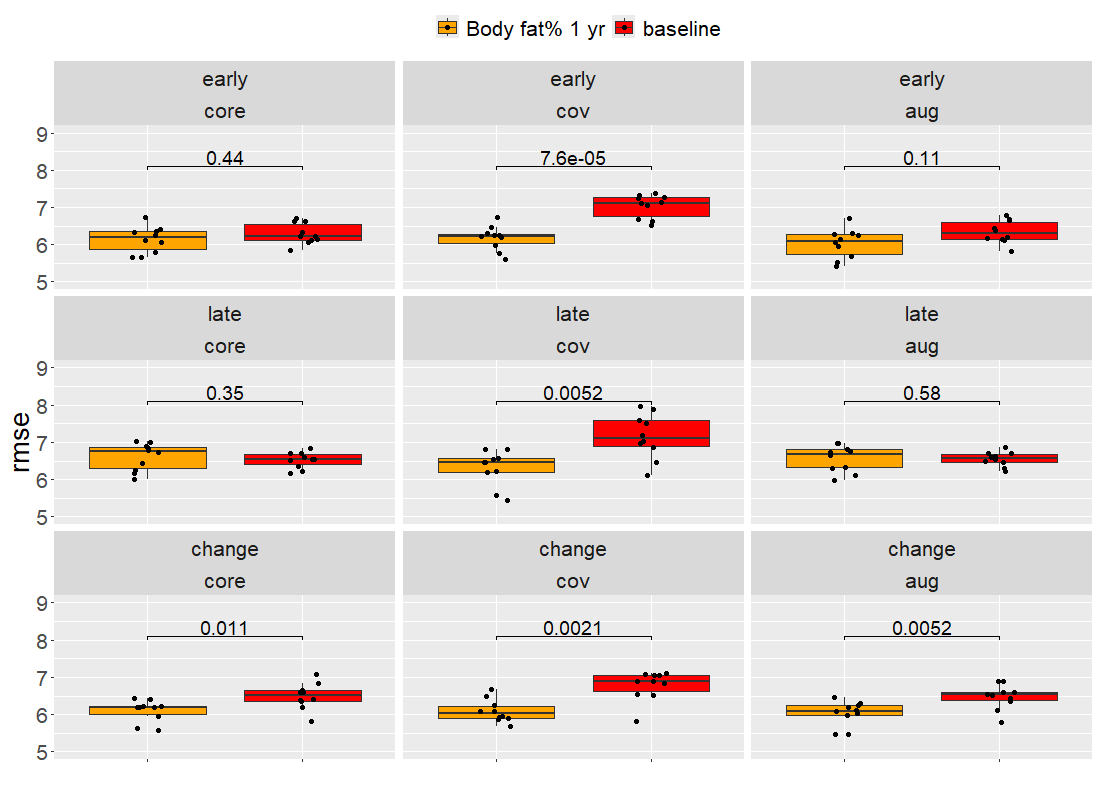
Supplemental Figure 16. The prediction performance of body fat% quantified by AUROC at one year postpartum with pathways in early and late pregnancy and with its change. Change in microbiota (p=0.01, Wilcoxon rank-sum test) and when combined with covariates (p=0.005, Wilcoxon rank-sum test) predicted body fat% at one year postpartum.

a)


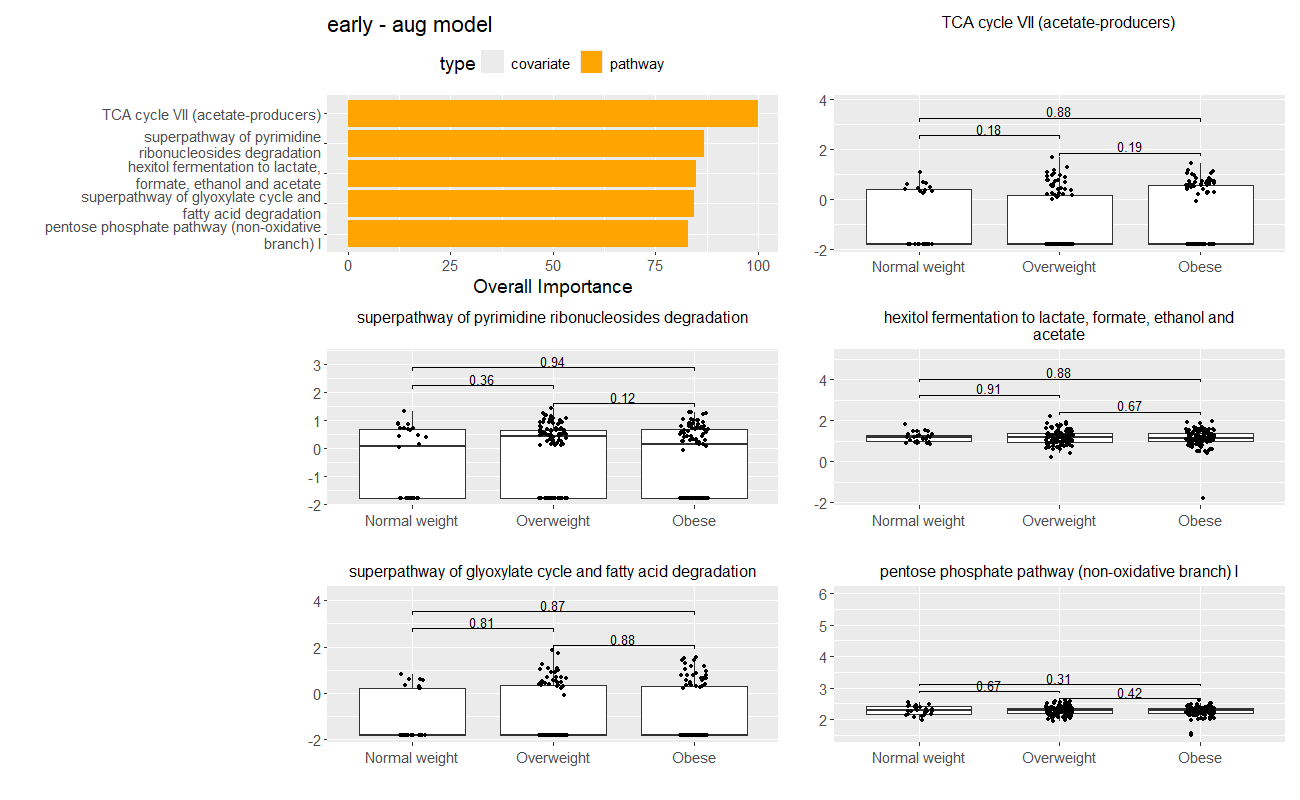


b)


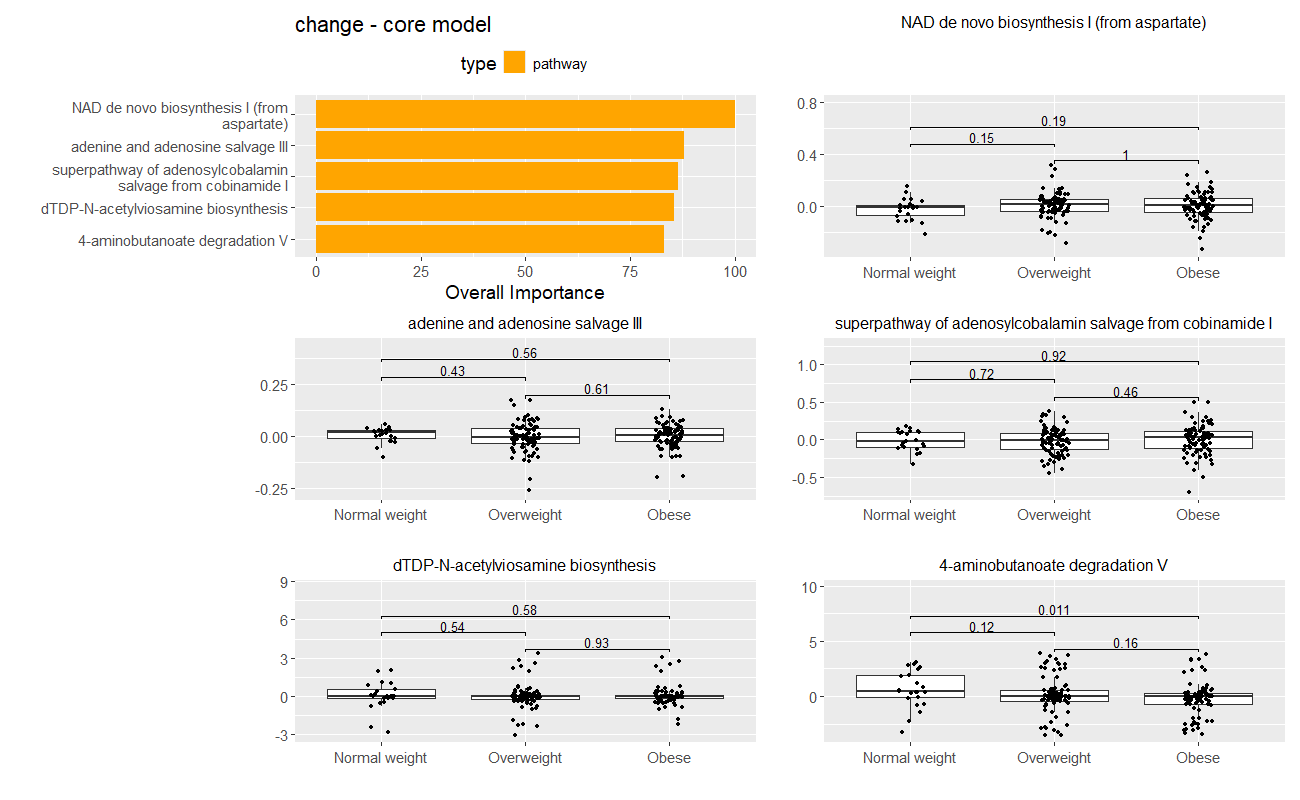


c)


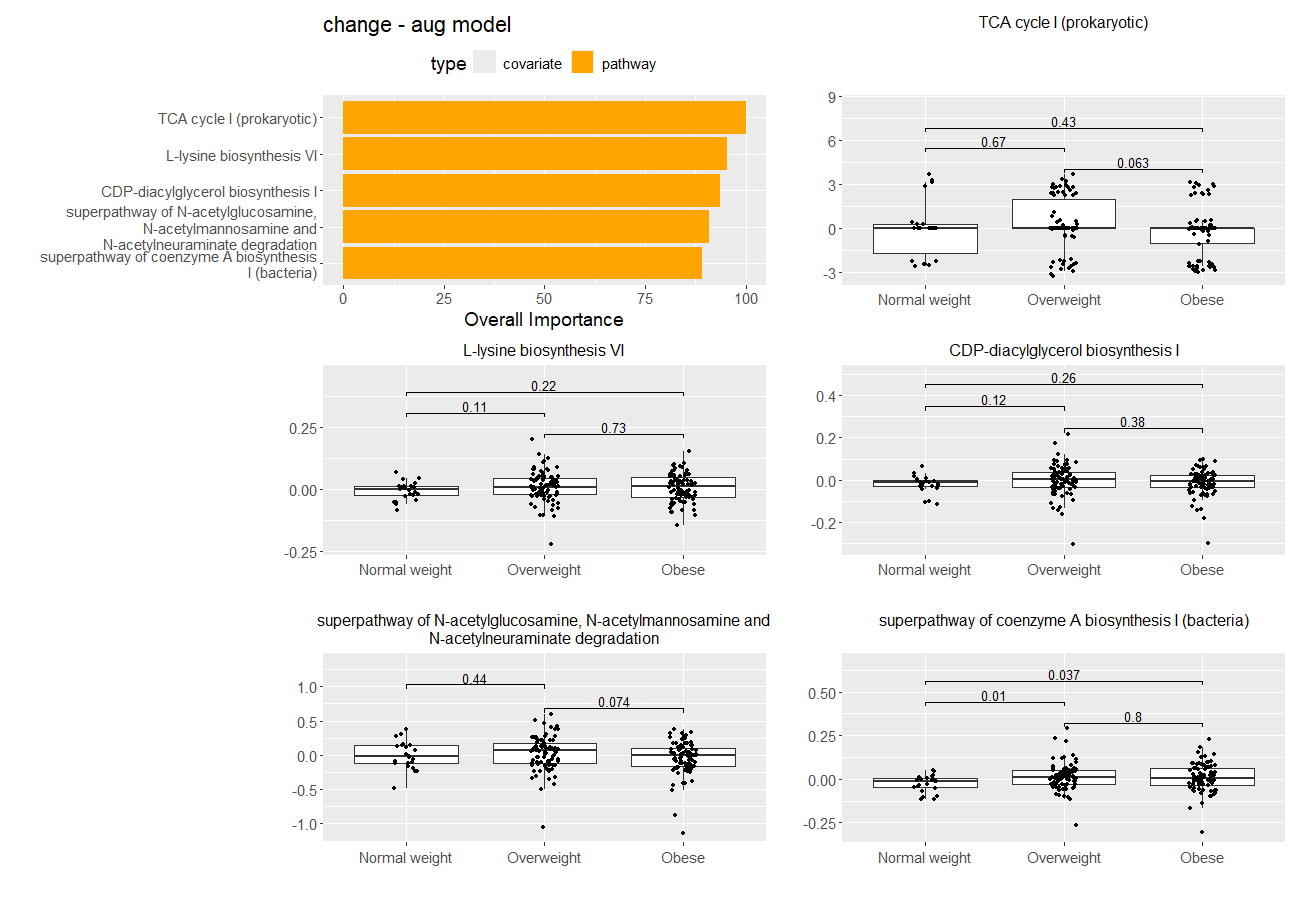


Supplemental Figure 17a-c. The pathways in early pregnancy (a) and the change from early to late pregnancy (b-c) predicting the BMI. Testing between the groups is done with the Wilcoxon rank-sum test and the statistical difference is depicted with a p-value.


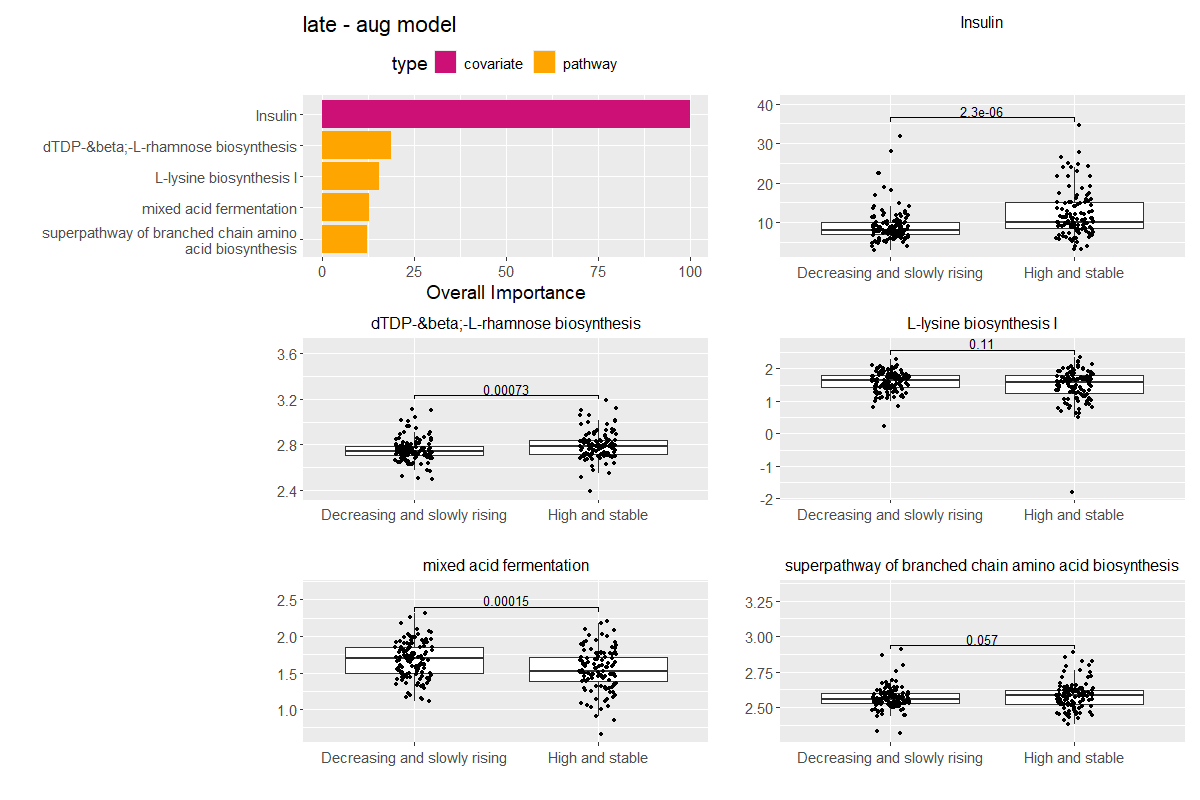


Supplemental Figure 18. The pathways in late pregnancy predicting the body fat% trajectories. Testing between the groups is done with the Wilcoxon rank-sum test and the statistical difference is depicted with a p-value.

a)


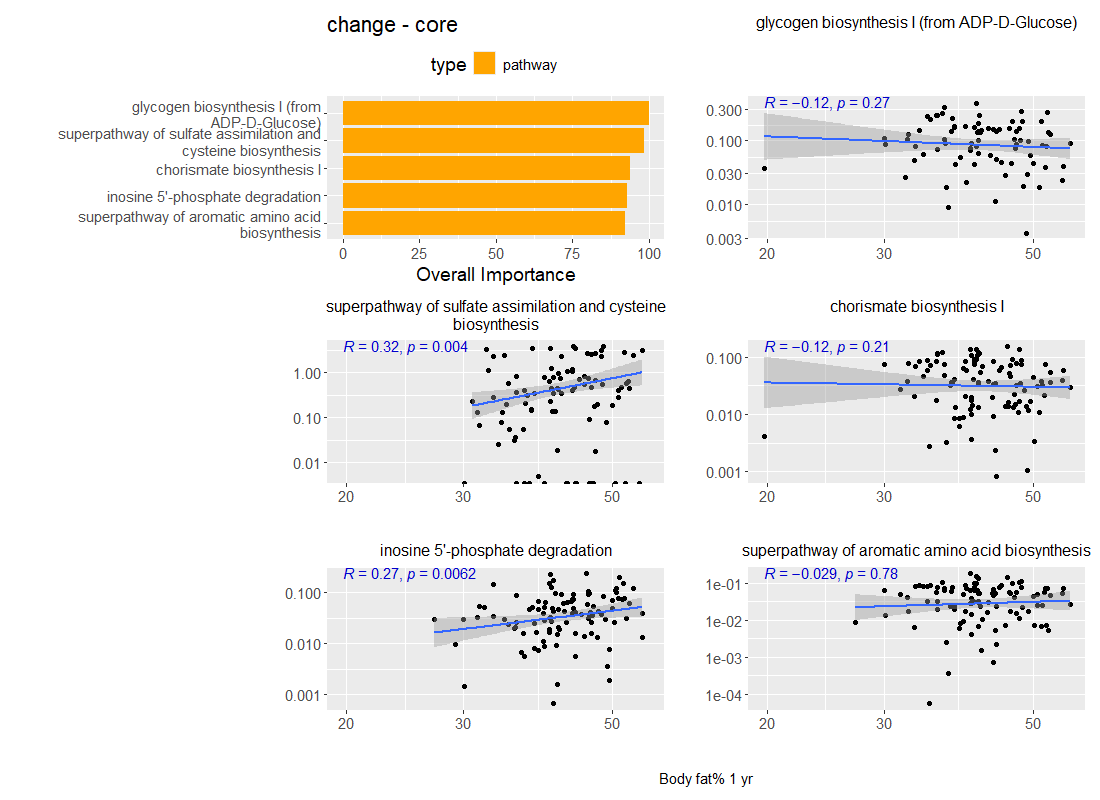


b)


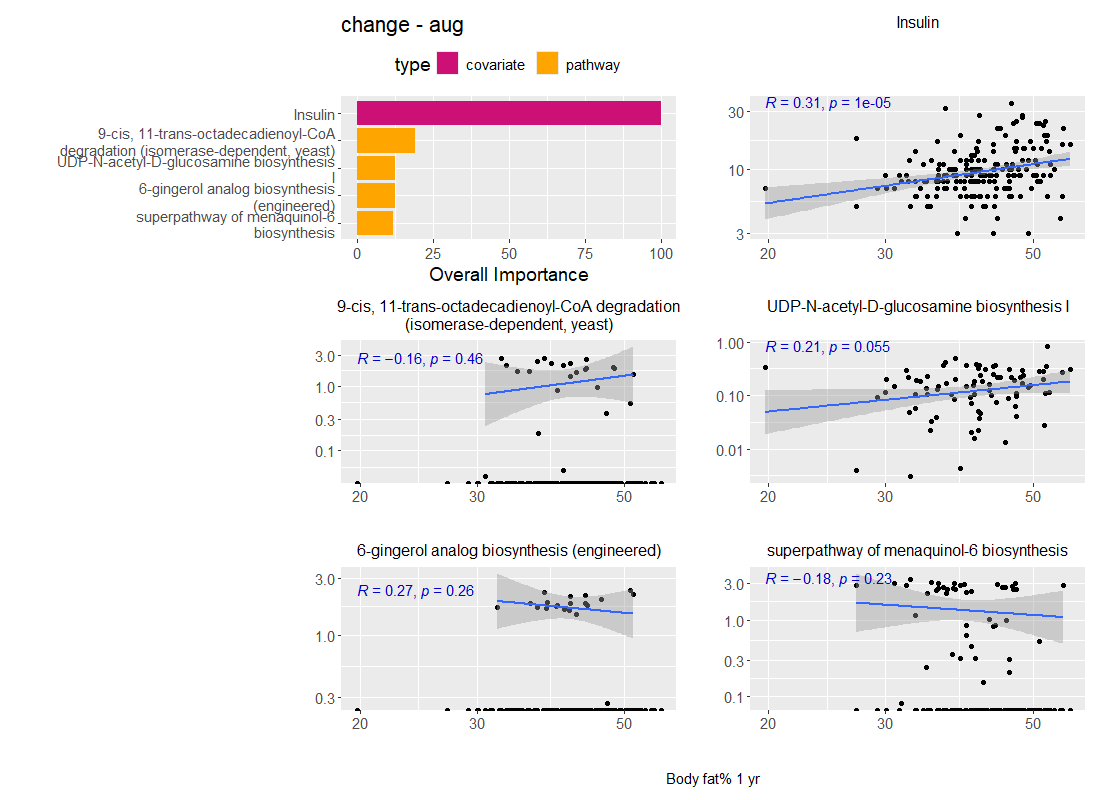


Supplemental Figure 19a-b. The change from early to late pregnancy (a-b) in the pathways predicting body fat%. The test is linear regression and the statistical difference is depicted with a p-value.
